# Supplementary material for: Win-win opportunities combining high yields with high multi-taxa biodiversity in tropical agroforestry
Source: Nat Commun. 2022 Jul 26;13:4127. doi: 10.1038/s41467-022-30866-8 (PMC9325886; doi:10.1038/s41467-022-30866-8)
Supplement: Supplementary file 1 — Supplementary Information [file 41467_2022_30866_MOESM1_ESM.pdf]

Supplementary Information

Manuscript title: Win-win opportunities combining high yields with high multi-taxa biodiversity in tropical agroforestry

Table of contents:

Number	Content
Supplementary Table 1	Effect of vanilla yield kg/ha on (endemic) mean normalized richness in interaction with land-use history
Supplementary Table 2	Effect of vanilla yield kg/ha on (endemic) species richness in interaction with land-use history
Supplementary Table 3	Species richness comparison between old-growth forest & forest-derived vanilla agroforest
Supplementary Table 4	Tukey multiple comparisons of means for all land-use-pairs of mean normalized richness
Supplementary Table 5	Test results of Kruskal-Wallis test and pairwise Wilcoxon-test for all land-use-pairs
Supplementary Table 6	Tukey multiple comparisons of means for all land-use-pairs of species richness
Supplementary Table 7	Alpha, gamma & beta diversity
Supplementary Table 8	Species richness comparison between forest fragment & forest-derived vanilla agroforest
Supplementary Table 9	Species richness comparison between fallow & fallow-derived vanilla agroforest
Supplementary Table 10	Sampling coverage for each taxon
Supplementary Table 11	Species composition - pairwise comparisons
Supplementary Table 12	Overview of vanilla plant/yield/agroforest statistics
Supplementary Table 13	Determinants of vanilla yield (model comparison)
Supplementary Table 14	Determinants of vanilla yield (final model)
Supplementary Table 15	Determinants of species richness (model comparison)
Supplementary Table 16	Determinants of endemic species richness (model comparison)
Supplementary Table 17	Determinants of species richness in 30 vanilla agroforests (final model)
Supplementary Table 18	Determinants of endemic species richness in 30 vanilla agroforests (final model)
Supplementary Table 19	Icon attribution
Supplementary Table 20	Mean, standard deviation, min and max value of all environmental and management variables
Supplementary Figure 1	Study design overview

Supplementary Figure 2	Exemplary photos of land-use types
Supplementary Figure 3	Species accumulation curves
Supplementary Figure 4	Species composition of the five land-use types
Supplementary Figure 5	Vanilla yield kg/ha depending on environmental and management variables
Supplementary Figure 6	Vanilla yield depending on land-use history
Supplementary Figure 7	(Endemic) species richness depending on environmental and management variables
Supplementary Figure 8	Vanilla pied design on plot
Supplementary Figure 9	Correlation matrix for all pairs of soil variables
Supplementary Figure 10	PCA based on eight soil characteristics
Supplementary Figure 11	Correlation matrix for all pairs of environmental and management variables
Supplementary Figure 12	Pollination labour input predicted by number of household members.

Supplementary Table 1: Linear mixed effect model (LMM) analyzing the effect of vanilla yield kg/ha (scaled & sqrt transformed) in interaction with land-use history on 1) mean normalized richness (trees (n=28), herbaceous plants (n=30), birds (n=30), amphibians (n=30), reptiles (n=30), butterflies (n=30) and ants (n=30)) and 2) mean normalized endemic richness (endemic richness of trees (n=28), herbaceous plants (n=30), birds (n=30), amphibians (n=30), reptiles (n=30), butterflies (n=30) and ants (n=30)). The p-values are based on a two-sided t-test testing whether the mean is different from 0. Significant effects are highlighted in bold. Digits were rounded to the third decimal place. Data used from 30 vanilla agroforests (20 fallow-derived vanilla agroforests, 10 forest-derived vanilla agroforests).

**1) Mean normalized richness**	**Estimate**	**Std. Error**	**df**	**t-value**	**p-value**
Intercept	0.131	0.005	11.020	24.603	<0.001
Vanilla yield kg/ha	-0.007	0.005	25.783	-1.425	0.166
Land-use history (VFOR)	0.047	0.009	25.945	5.077	**<0.001**
Vanilla yield kg/ha: Land-use history	-0.013	0.012	25.784	-1.043	0.307
**2) Mean normalized endemic richness**	**Estimate**	**Std. Error**	**df**	**t-value**	**p-value**
Intercept	0.059	0.006	12.214	9.098	<0.001
Vanilla yield kg/ha	-0.001	0.005	25.609	-0.201	0.842
Land-use history (VFOR)	0.061	0.010	25.783	6.183	**<0.001**
Vanilla yield kg/ha : Land-use history	-0.012	0.013	24.318	-0.931	0.361

Supplementary Table 2: General linear mixed effect models (glmmTMB) analyzing the effect of vanilla yield kg/ha (scaled & sqrt transformed) in interaction with land-use history on species richness across taxa: (endemic) trees (n=28), (endemic) herbaceous plants (n=30), (endemic) birds (n=30), (endemic) amphibians (n=30), (endemic) reptiles (n=30), (endemic) butterflies (n=30) and (endemic) ants (n=30). The p-values are based on two-sided t-tests testing whether the mean is different from 0. Significant effects are highlighted in bold. Digits were rounded to the third decimal place. Data used from 30 vanilla agroforests (20 fallow-derived vanilla agroforests, 10 forest-derived vanilla agroforests).

	**Estimate**	**Std. Error**	**z-value**	**p-value**
**Tree richness**				
Intercept	2.482	0.126	19.752	<0.001
Vanilla yield kg/ha	-0.051	0.075	-0.677	0.498
Land-use history (VFOR)	0.921	0.112	8.199	**<0.001**
Vanilla yield kg/ha : Land-use history	-0.144	0.118	-1.219	0.223
**Herbs richness**				
Intercept	2.988	0.124	24.022	<0.001
Vanilla yield kg/ha	-0.077	0.076	-1.003	0.316
Land-use history (VFOR)	0.008	0.151	0.052	0.959
Vanilla yield kg/ha : Land-use history	0.083	0.201	0.410	0.682
**Bird richness**				
Intercept	1.843	0.064	28.875	<0.001
Vanilla yield kg/ha	-0.032	0.057	-0.560	0.575
Land-use history (VFOR)	0.188	0.098	1.911	0.056
Vanilla yield kg/ha : Land-use history	-0.037	0.129	-0.287	0.774
**Amphibians richness**				
Intercept	1.514	0.062	24.448	< 0.001
Vanilla yield kg/ha	0.110	0.055	2.001	**0.045**
Land-use history (VFOR)	0.013	0.108	0.122	0.903
Vanilla yield kg/ha : Land-use history	-0.120	0.142	-0.840	0.401
**Reptile richness**				
Intercept	1.908	0.090	21.280	<0.001
Vanilla yield kg/ha	-0.125	0.082	-1.512	0.131
Land-use history (VFOR)	0.303	0.142	2.140	**0.032**
Vanilla yield kg/ha : Land-use history	0.282	0.175	1.615	0.106
**Butterflies richness**				
Intercept	2.329	0.085	27.258	< 0.001
Vanilla yield kg/ha	-0.179	0.071	-2.525	**0.012**
Land-use history (VFOR)	0.073	0.196	0.372	0.710
Vanilla yield kg/ha : Land-use history	0.220	0.202	1.088	0.277
**Ants richness**				
Intercept	2.737	0.063	43.390	< 0.001
Vanilla yield kg/ha	0.010	0.057	0.180	0.858
Land-use history (VFOR)	0.177	0.099	1.790	0.073
Vanilla yield kg/ha : Land-use history	-0.093	0.131	-0.710	0.478
**Endemic Tree richness**				
Intercept	0.607	0.359	1.692	0.091

Vanilla yield kg/ha	0.224	0.188	1.191	0.234
Land-use history (VFOR)	2.141	0.236	9.074	**< 0.001**
Vanilla yield kg/ha : Land-use history	-0.366	0.214	-1.712	0.087
**Endemic Herbaceous plant richness**				
Intercept	0.895	0.106	8.414	<0.001
Vanilla yield kg/ha	0.107	0.095	1.123	0.261
Land-use history (VFOR)	0.456	0.160	2.858	**0.004**
Vanilla yield kg/ha : Land-use history	-0.033	0.198	-0.168	0.867
**Endemic Birds richness**				
Intercept	-0.232	0.261	-0.892	0.373
Vanilla yield kg/ha	-0.300	0.240	-1.254	0.210
Land-use history (VFOR)	1.078	0.334	3.227	**0.001**
Vanilla yield kg/ha : Land-use history	0.145	0.402	0.361	0.718
**Endemic Amphibian richness**				
Intercept	1.328	0.116	11.476	<0.001
Vanilla yield kg/ha	0.164	0.101	1.623	0.105
Land-use history (VFOR)	0.063	0.197	0.318	0.750
Vanilla yield kg/ha : Land-use history	-0.204	0.260	-0.783	0.434
**Endemic Reptile richness**				
Intercept	1.628	0.094	17.254	< 0.001
Vanilla yield kg/ha	-0.145	0.060	-2.401	**0.016**
Land-use history (VFOR)	0.074	0.118	0.626	0.531
Vanilla yield kg/ha : Land-use history	0.289	0.132	2.197	**0.028**
**Endemic Butterflies richness**				
Intercept	1.852	0.056	33.060	< 0.001
Vanilla yield kg/ha	-0.104	0.051	-2.020	**0.043**
Land-use history (VFOR)	0.072	0.095	0.760	0.447
Vanilla yield kg/ha : Land-use history	0.038	0.126	0.300	0.763
**Endemic Ants richness**				
Intercept	1.161	0.154	7.560	<0.001
Vanilla yield kg/ha	0.116	0.128	0.910	0.363
Land-use history (VFOR)	0.717	0.195	3.680	**<0.001**
Vanilla yield kg/ha : Land-use history	-0.063	0.238	-0.265	0.791

Supplementary Table 3: Mean values of (endemic) mean normalized richness and (endemic) species richness of seven taxa in forest-derived vanilla agroforest (**VFOR**) and old-growth forest (**FOR**) with absolute difference (mean richness VFOR- mean richness FOR) and relative percentage difference (=mean richness VFOR*100/mean richness FOR) and relative percentage gain or loss from old-growth forest to forest-derived vanilla agroforest (=absolute difference*100/mean richness FOR). Relative percentage loss is highlighted in yellow.

Land-use type	FOR	VFOR	Absolute difference FOR-VFOR	Relative difference % VFOR-FOR	Relative Gain/Loss in %
Mean normalized richness	0.23	0.18	-0.052	77	-23
Mean normalized endemic richness	0.22	0.12	-0.105	53	-47
Birds	12.30	7.60	-4.700	62	-38
Endemic Birds	7.40	2.30	-5.100	31	-69
Herbs	15.50	20.60	5.100	133	33
Endemic herbs	5.50	3.90	-1.600	71	-29
Amphibians	9.30	4.60	-4.700	49	-51
Endemic amphibians	9.30	4.00	-5.300	43	-57
Reptiles	9.80	9.40	-0.400	96	-4
Endemic reptiles	8.50	5.90	-2.600	69	-31
Butterflies	6.20	11.30	5.100	182	82
Endemic butterflies	6.10	6.80	0.700	111	11
Ants	16.40	18.40	2.000	112	12
Endemic ants	9.60	6.90	-2.700	72	-28
Tree	64.40	31.30	-33.100	49	-51
Endemic trees	47.30	19.90	-27.400	42	-58

Supplementary Table 4: Tukey multiple comparison test of means for all land-use-pairs of **mean normalized richness** (including 7 taxa: tree, herbaceous plant, birds, reptiles, amphibians, butterflies and ants). Pairwise comparisons calculated with the glht-function of the package 'multcomp' with all terms significant at <0.05 (highlighted in bold). The test compares the difference between each pair of means with bonferroni adjustment. Linear-mixed effect models (LMM) were used. FOR=Old-growth forest; FFOR= Forest fragment; VFOR=Forest-derived vanilla agroforest; VFAL=Fallow-derived vanilla agroforest; FAL=Fallow.

**Mean normalized richness**	**Estimate**	**Std. Error**	**z-value**	**p-value**
FFOR-FOR	-0.062	0.012	-5.202	**<0.001**
VFOR-FOR	-0.052	0.012	-4.360	**<0.001**
FAL-FOR	-0.111	0.010	-10.579	**<0.001**
VFAL-FOR	-0.096	0.010	-9.207	**<0.001**
VFOR-FFOR	0.010	0.012	0.846	0.915
FAL-FFOR	-0.048	0.010	-4.733	**<0.001**
VFAL-FFOR	-0.034	0.010	-3.329	**0.008**
FAL-VFOR	-0.058	0.010	-5.693	**<0.001**
VFAL-VFOR	-0.044	0.010	-4.278	**<0.001**
VFAL-FAL	0.014	0.008	1.714	0.421

Supplementary Table 5: Test results of Kruskal-Wallis test and pairwise Wilcoxon-test for all land-use-pairs of **mean normalized endemic richness** (including 7 taxa: tree, herbaceous plant, birds, reptiles, amphibians, butterflies and ants) and (endemic) tree richness. Non-parametric Kruskal–Wallis test, followed by pairwise Wilcoxon test was used to account for heteroscedastic residuals for these taxa. FOR=Old-growth forest; FFOR= Forest fragment; VFOR=Forest-derived vanilla agroforest; VFAL=Fallow-derived vanilla agroforest; FAL=Fallow. Significant terms at <0.05 are highlighted in bold.

**Mean normalized endemic richness**				
Kruskal-Wallis	χ^2=54.363; Df=4; p-value= **<0.001**			
	FOR	FFOR	VFOR	FAL
FFOR	**0.005**	-	-	-
VFOR	**0.002**	1.000	-	-
FAL	**<0.001**	**<0.001**	**0.001**	-
VFAL	**<0.001**	**0.001**	**0.002**	**0.002**
**Tree richness**				
Kruskal-Wallis	χ^2=52.42; Df=4; p-value= **<0.001**			
	FOR	FFOR	VFOR	FAL
FFOR	0.100	-	-	-
VFOR	**0.015**	1.000	-	-
FAL	**<0.001**	**<0.001**	**<0.001**	-
VFAL	**<0.001**	**<0.001**	**0.014**	**0.003**
**Endemic tree richness**				
Kruskal-Wallis	χ^2=48.612; Df=4; p-value= **<0.001**			
	FOR	FFOR	VFOR	FAL
FFOR	0.090	-	-	-
VFOR	**0.015**	1.000	-	-
FAL	**<0.001**	**<0.001**	**0.002**	-
VFAL	**<0.001**	**<0.001**	**0.006**	0.690

Supplementary Table 6: Tukey multiple comparison test of means for all land-use-pairs showing dissimilarities among land-use systems of (endemic) tree, (endemic) herbaceous plant, (endemic) bird, (endemic) amphibian, (endemic) reptile, (endemic) butterfly and (endemic) ant richness. The test compares the difference between each pair of means with bonferroni adjustment. Pairwise comparisons calculated with the glht-function of the package 'multcomp' including Bonferroni correction with all terms significant at <0.05 (highlighted in bold). FOR=Old-growth forest; FFOR= Forest fragment; VFOR=Forest-derived vanilla agroforest; VFAL=Fallow-derived vanilla agroforest; FAL=Fallow.

**Herbaceous plant richness**	**Estimate**	**Std. Error**	**z-value**	**p-value**
FFOR-FOR	-0.099	0.208	-0.477	0.989
VFOR-FOR	0.285	0.204	1.397	0.623
FAL-FOR	0.517	0.180	2.867	**0.032**
VFAL-FOR	0.292	0.182	1.604	0.488
VFOR-FFOR	0.385	0.189	2.030	0.246
FAL-FFOR	0.616	0.163	3.770	**0.001**
VFAL-FFOR	0.392	0.165	2.381	0.117
FAL-VFOR	0.232	0.157	1.472	0.574
VFAL-VFOR	0.007	0.162	0.045	1.000
VFAL-FAL	-0.224	0.129	-1.737	0.404
**Endemic herbaceous plant richness**	**Estimate**	**Std. Error**	**z-value**	**p-value**
FFOR-FOR	-0.223	0.202	-1.103	0.805
VFOR-FOR	-0.344	0.209	-1.642	0.470
FAL-FOR	-0.894	0.201	-4.447	**<0.001**
VFAL-FOR	-0.809	0.196	-4.117	**<0.001**
VFOR-FFOR	-0.121	0.220	-0.548	0.982
FAL-FFOR	-0.671	0.212	-3.163	**0.013**
VFAL-FFOR	-0.586	0.208	-2.819	**0.039**
FAL-VFOR	-0.550	0.219	-2.514	0.087
VFAL-VFOR	-0.465	0.215	-2.166	0.192
VFAL-FAL	0.085	0.206	0.412	0.994
**Bird richness**	**Estimate**	**Std. Error**	**z-value**	**p-value**
FFOR-FOR	-0.578	0.150	-3.843	**0.001**
VFOR-FOR	-0.481	0.146	-3.300	**0.009**
FAL-FOR	-0.669	0.127	-5.278	**<0.001**
VFAL-FOR	-0.661	0.127	-5.226	**<0.001**
VFOR-FFOR	0.097	0.166	0.581	0.978
FAL-FFOR	-0.091	0.150	-0.607	0.974
VFAL-FFOR	-0.083	0.150	-0.555	0.981
FAL-VFOR	-0.188	0.145	-1.292	0.694
VFAL-VFOR	-0.180	0.145	-1.239	0.726
VFAL-FAL	0.008	0.126	0.063	1.000
**Endemic bird richness**	**Estimate**	**Std. Error**	**z-value**	**p-value**
FFOR-FOR	-1.307	0.269	-4.858	**<0.001**
VFOR-FOR	-1.168	0.259	-4.514	**<0.001**
FAL-FOR	-2.106	0.279	-7.542	**<0.001**

VFAL-FOR	-2.162	0.285	-7.576	**<0.001**
VFOR-FFOR	0.139	0.307	0.453	0.991
FAL-FFOR	-0.799	0.325	-2.458	0.099
VFAL-FFOR	-0.855	0.330	-2.590	0.071
FAL-VFOR	-0.938	0.316	-2.965	**0.025**
VFAL-VFOR	-0.995	0.324	-3.075	**0.018**
VFAL-FAL	-0.057	0.339	-0.168	1.000
**Amphibian richness**	**Estimate**	**Std. Error**	**z-value**	**p-value**
FFOR-FOR	-0.601	0.134	-4.494	**<0.001**
VFOR-FOR	-0.704	0.139	-5.080	**<0.001**
FAL-FOR	-0.977	0.122	-8.005	**<0.001**
VFAL-FOR	-0.715	0.113	-6.319	**<0.001**
VFOR-FFOR	-0.103	0.157	-0.658	0.965
FAL-FFOR	-0.376	0.142	-2.644	0.062
VFAL-FFOR	-0.114	0.135	-0.846	0.915
FAL-VFOR	-0.273	0.147	-1.859	0.337
VFAL-VFOR	-0.011	0.140	-0.078	1.000
VFAL-FAL	0.262	0.123	2.128	0.206
**Endemic amphibian richness**	**Estimate**	**Std. Error**	**z-value**	**p-value**
FFOR-FOR	-0.601	0.133	-4.531	**<0.001**
VFOR-FOR	-0.844	0.145	-5.834	**<0.001**
FAL-FOR	-1.200	0.130	-9.209	**<0.001**
VFAL-FOR	-0.895	0.118	-7.580	**<0.001**
VFOR-FFOR	-0.243	0.162	-1.501	0.558
FAL-FFOR	-0.600	0.149	-4.017	**<0.001**
VFAL-FFOR	-0.294	0.139	-2.121	0.208
FAL-VFOR	-0.357	0.160	-2.228	0.167
VFAL-VFOR	-0.051	0.150	-0.341	0.997
VFAL-FAL	0.305	0.137	2.236	0.164
**Reptile richness**	**Estimate**	**Std. Error**	**z-value**	**p-value**
FFOR-FOR	-0.022	0.162	-0.138	1.000
VFOR-FOR	-0.052	0.165	-0.317	0.998
FAL-FOR	-0.685	0.161	-4.242	**<0.001**
VFAL-FOR	-0.355	0.153	-2.324	0.136
VFOR-FFOR	-0.030	0.147	-0.203	1.000
FAL-FFOR	-0.662	0.143	-4.624	**<0.001**
VFAL-FFOR	-0.333	0.134	-2.490	0.092
FAL-VFOR	-0.632	0.146	-4.321	**<0.001**
VFAL-VFOR	-0.303	0.140	-2.171	0.189
VFAL-FAL	0.329	0.133	2.484	0.093
**Endemic reptile richness**	**Estimate**	**Std. Error**	**z-value**	**p-value**
FFOR-FOR	-0.149	0.168	-0.883	0.901
VFOR-FOR	-0.370	0.176	-2.096	0.215
FAL-FOR	-0.815	0.169	-4.837	**<0.001**
VFAL-FOR	-0.494	0.163	-3.029	**0.020**
VFOR-FFOR	-0.221	0.129	-1.717	0.415
FAL-FFOR	-0.667	0.118	-5.669	**<0.001**

VFAL-FFOR	-0.346	0.110	-3.150	**0.014**
FAL-VFOR	-0.446	0.129	-3.457	**0.0048**
VFAL-VFOR	-0.125	0.126	-0.993	0.855
VFAL-FAL	0.321	0.110	2.916	**0.028**
**Butterfly richness**	**Estimate**	**Std. Error**	**z-value**	**p-value**
FFOR-FOR	-0.198	0.212	-0.933	0.878
VFOR-FOR	0.551	0.190	2.905	**0.028**
FAL-FOR	0.470	0.175	2.691	0.052
VFAL-FOR	0.548	0.175	3.135	**0.014**
VFOR-FFOR	0.749	0.173	4.335	**<0.001**
FAL-FFOR	0.668	0.157	4.258	**<0.001**
VFAL-FFOR	0.746	0.157	4.741	**<0.001**
FAL-VFOR	-0.081	0.124	-0.654	0.964
VFAL-VFOR	-0.003	0.130	-0.022	1.000
VFAL-FAL	0.078	0.101	0.771	0.935
**Endemic butterfly richness**	**Estimate**	**Std. Error**	**z-value**	**p-value**
FFOR-FOR	-0.327	0.170	-1.922	0.300
VFOR-FOR	0.109	0.151	0.720	0.951
FAL-FOR	-0.179	0.139	-1.290	0.693
VFAL-FOR	0.056	0.133	0.419	0.993
VFOR-FFOR	0.435	0.166	2.620	0.065
FAL-FFOR	0.148	0.155	0.951	0.874
VFAL-FFOR	0.382	0.150	2.548	0.078
FAL-VFOR	-0.288	0.134	-2.145	0.198
VFAL-VFOR	-0.053	0.128	-0.412	0.994
VFAL-FAL	0.235	0.114	2.067	0.230
**Ant richness**	**Estimate**	**Std. Error**	**z-value**	**p-value**
FFOR-FOR	-0.070	0.121	-0.576	0.978
VFOR-FOR	0.117	0.117	0.999	0.854
FAL-FOR	-0.214	0.109	-1.960	0.282
VFAL-FOR	-0.061	0.107	-0.572	0.979
VFOR-FFOR	0.187	0.110	1.691	0.435
FAL-FFOR	-0.144	0.102	-1.417	0.613
VFAL-FFOR	0.009	0.099	0.087	1.000
FAL-VFOR	-0.331	0.097	-3.405	**0.006**
VFAL-VFOR	-0.178	0.096	-1.861	0.335
VFAL-FAL	0.152	0.084	1.814	0.361
**Endemic ant richness**	**Estimate**	**Std. Error**	**z-value**	**p-value**
FFOR-FOR	-0.375	0.179	-2.095	0.218
VFOR-FOR	-0.339	0.180	-1.885	0.320
FAL-FOR	-2.039	0.239	-8.548	**<0.001**
VFAL-FOR	-1.064	0.180	-5.924	**<0.001**
VFOR-FFOR	0.036	0.175	0.206	1.000
FAL-FFOR	-1.664	0.235	-7.085	**<0.001**
VFAL-FFOR	-0.689	0.175	-3.942	**0.001**
FAL-VFOR	-1.700	0.235	-7.223	**<0.001**
VFAL-VFOR	-0.725	0.178	-4.073	**<0.001**

VFAL-FAL	0.975	0.235	4.143	**<0.001**

Supplementary Table 7: Table of alpha diversity (mean species richness across all plot of each land-use type) and gamma diversity (sum of species richness of all plots of each land-use type). For gamma diversity: 10 plots of each land-use type were randomly selected (Note: 5 plots of herbaceous fallow and 5 plots of woody fallow = 10 fallow plots).

	FOR	FFOR	VFOR	FLW	VFAL
**Mean normalized alpha richness**	0.23	0.17	0.18	0.12	0.13
**Mean normalized alpha endemic richness**	0.22	0.14	0.12	0.05	0.06
**Alpha Tree richness**	64.40	41.10	31.30	10.20	12.72
**Gamma Tree richness**	257.00	230.00	198.00	51.00	69.00
**Alpha Endemic Tree richness**	47.30	27.70	19.90	3.60	2.50
**Gamma Endemic Tree richness**	180.00	152.00	125.00	21.00	21.00
**Alpha Herb richness**	15.50	14.10	20.60	25.80	21.05
**Gamma Herb richness**	77.00	75.00	92.00	108.00	92.00
**Alpha Endemic Herb richness**	5.50	4.40	3.90	2.25	2.45
**Gamma Endemic Herb richness**	24.00	27.00	19.00	10.00	12.00
**Alpha Bird richness**	12.30	6.90	7.60	6.30	6.35
**Gamma Bird richness**	34.00	23.00	25.00	18.00	18.00
**Alpha Endemic Bird richness**	7.40	2.00	2.30	1.00	0.85
**Gamma Endemic Bird richness**	24.00	10.00	13.00	6.00	7.00
**Alpha Amphibian richness**	9.30	5.10	4.60	3.50	4.55
**Gamma Amphibian richness**	32.00	26.00	14.00	6.00	12.00
**Alpha Endemic Amphibian richness**	9.30	5.10	4.00	2.80	3.80
**Gamma Endemic Amphibian richness**	32.00	26.00	13.00	5.00	11.00
**Alpha Reptile richness**	19.10	14.70	14.00	8.45	11.40
**Gamma Reptile richness**	66.00	56.00	44.00	23.00	34.00
**Alpha Endemic Reptile richness**	9.30	5.10	4.00	2.80	3.80
**Gamma Endemic Reptile richness**	32.00	26.00	13.00	5.00	11.00
**Alpha Butterfly richness**	6.20	5.10	11.30	9.95	10.50
**Gamma Butterfly richness**	20.00	19.00	44.00	39.00	35.00
**Alpha Endemic Butterfly richness**	6.10	4.40	6.80	5.10	6.45
**Gamma Endemic Butterfly richness**	19.00	14.00	24.00	19.00	21.00
**Alpha Ant richness**	16.40	15.30	18.40	13.25	15.45
**Gamma Ant richness**	55.00	58.00	71.00	47.00	54.00
**Alpha Endemic Ant richness**	9.60	6.60	6.90	2.27	3.67
**Gamma Endemic Ant richness**	32.00	28.00	32.00	10.00	20.00

Supplementary Table 8: Mean values of (endemic) mean normalized richness and (endemic) species richness of seven taxa in forest-derived vanilla agroforest (**VFOR**) and forest fragment (**FFOR**) with absolute difference (mean richness VFOR- mean richness FFOR) and relative percentage difference (=mean richness VFOR*100/mean richness FFOR) and relative percentage gain or loss from forest fragment to forest-derived vanilla agroforest (=absolute difference*100/mean richness FFOR). Relative percentage loss is highlighted in yellow

Land-use type	FFOR	VFOR	Absolute difference FFOR-VFOR	Relative difference % VFOR-FFOR	Relative Gain/Loss in %
Mean normalized richness	0.17	0.18	0.010	106	6
Mean normalized endemic richness	0.14	0.12	-0.019	86	-14
Birds	6.90	7.60	0.700	110	10
Endemic Birds	2.00	2.30	0.300	115	15
Herbs	14.10	20.60	6.500	146	46
Endemic herbs	4.40	3.90	-0.500	89	-11
Amphibians	5.10	4.60	-0.500	90	-10
Endemic amphibians	5.10	4.00	-1.100	78	-22
Reptiles	9.60	9.40	-0.200	98	-2
Endemic reptiles	7.40	5.90	-1.500	80	-20
Butterflies	5.10	11.30	6.200	222	122
Endemic butterflies	4.40	6.80	2.400	155	55
Ants	15.30	18.40	3.100	120	20
Endemic ants	6.60	6.90	0.300	105	5
Tree	41.10	31.30	-9.800	76	-24
Endemic trees	27.70	19.90	-7.800	72	-28

Supplementary Table 9: Mean values of (endemic) mean normalized richness and (endemic) species richness of seven taxa in fallow-derived vanilla agroforest (**VFAL**) and fallows (**FAL**) with the absolute difference (mean richness VFAL- mean richness FAL) and relative percentage difference (=mean richness VFAL*100/mean richness FAL) and relative percentage gain or loss from fallow to fallow-derived vanilla agroforest (=absolute difference*100/mean richness fallow). Relative percentage gain is highlighted in blue

Land-use type	FAL	VFAL	Absolute difference FAL-VFAL	Relative difference % VFAL-FAL	Relative Gain/Loss in %
Mean normalized richness	0.12	0.13	0.014	112	12
Mean normalized endemic richness	0.04	0.06	0.016	138	38
Birds	6.30	6.35	0.050	101	1
Endemic Birds	0.90	0.85	-0.050	94	-6
Herbs	25.80	21.05	-4.750	82	-18
Endemic herbs	2.25	2.45	0.200	109	9
Amphibians	3.50	4.55	1.050	130	30
Endemic amphibians	2.80	3.80	1.000	136	36
Reptiles	4.95	6.85	1.900	138	38
Endemic reptiles	3.80	5.25	1.450	138	38
Butterflies	9.95	10.50	0.550	106	6
Endemic butterflies	5.10	6.45	1.350	126	26
Ants	13.25	15.45	2.200	117	17
Endemic ants	1.25	3.30	2.050	264	164
Tree	5.10	12.72	7.622	249	149
Endemic trees	1.80	2.50	0.700	139	39

Supplementary Table 10: Sampling coverage (SC) depending on taxon for each land-use type (FOR=Old-growth forest, FFOR= Forest fragment, VFOR=Forest-derived vanilla agroforest, FAL=Fallow, VFAL=Fallow-derived vanilla agroforest. Sampling computed with *iNext* function, Hill-number q=0, datatype=incidence raw, endpoint=20. Last row displays average values of sampling coverage for each taxon.

	SC Birds	SC Butterflies	SC Trees	SC Ants	SC Herbs	SC Reptiles	SC Amphibians
FOR	0.91	0.86	0.80	0.86	0.73	0.89	0.85
FFOR	0.86	0.81	0.70	0.80	0.69	0.87	0.67
VFOR	0.85	0.86	0.62	0.80	0.79	0.89	0.88
FAL	0.95	0.90	0.58	0.93	0.88	0.92	0.97
VFAL	0.95	0.91	0.87	0.92	0.86	0.91	0.89
Mean	0.90	0.87	0.71	0.86	0.79	0.90	0.85

Supplementary Table 11: Species composition multilevel pairwise comparisons of species richness of 7 taxa across land-use types (with False discovery rate correction) and results of multivariate dispersion test based on Jaccard dissimilarity matrix using the betadisp and the permutest function of the vegan package with false discovery rate. Significant p-values (<0.05) are highlighted with a star (*).

**Trees**	F Model	R^2	p-value	p-adjusted	sig	
VFOR vs. FFOR	0.778	0.041	0.970	0.970		
VFOR vs. VFAL	2.265	0.080	0.001	0.001	*	
VFOR vs. FAL	1.376	0.071	0.036	0.040	*	
VFOR vs. FOR	2.275	0.112	0.001	0.001	*	
FFOR vs. VFAL	3.449	0.117	0.001	0.001	*	
FFOR vs. FAL	2.077	0.103	0.001	0.001	*	
FFOR vs. FOR	1.946	0.098	0.001	0.001	*	
VFAL vs. FAL	1.601	0.058	0.010	0.013	*	
VFAL vs. FOR	5.840	0.183	0.001	0.001	*	
FAL vs. FOR	4.235	0.190	0.001	0.001	*	
	Df	Sums of Squares	Mean of squares	F-Model	R2	Pr(>F)
Groups	4.000	0.064	0.016	10.956	999.000	0.001 ***
Residuals	53.000	0.078	0.001			
**Herbs**	F Model	R^2	p-value	p-adjusted	sig	
VFOR vs. FFOR	2.445	0.120	0.001	0.001	*	
VFOR vs. VFAL	1.783	0.060	0.006	0.006	*	
VFOR vs. FAL	3.159	0.101	0.001	0.001	*	
VFOR vs. FOR	3.617	0.167	0.001	0.001	*	
FFOR vs. VFAL	4.595	0.141	0.001	0.001	*	
FFOR vs. FAL	4.549	0.140	0.001	0.001	*	
FFOR vs. FOR	2.159	0.107	0.001	0.001	*	
VFAL vs. FAL	2.953	0.072	0.001	0.001	*	
VFAL vs. FOR	5.525	0.165	0.001	0.001	*	

FAL vs. FOR	5.739	0.170	0.001	0.001	*	
	Df	Sums of Squares	Mean of squares	F-Model	R2	Pr(>F)
Groups	4.000	0.011	0.003	0.894	999.000	0.471
Residuals	65.000	0.191	0.003			
**Birds**	F Model	R^2	p-value	p-adjusted	sig	
VFOR vs. FFOR	0.403	0.022	0.974	0.974		
VFOR vs. VFAL	2.335	0.077	0.014	0.016	*	
VFOR vs. FAL	2.695	0.088	0.003	0.004	*	
VFOR vs. FOR	3.000	0.143	0.003	0.004	*	
FFOR vs. VFAL	2.782	0.090	0.001	0.003	*	
FFOR vs. FAL	2.918	0.094	0.004	0.005	*	
FFOR vs. FOR	3.424	0.160	0.001	0.003	*	
VFAL vs. FAL	2.798	0.069	0.002	0.004	*	
VFAL vs. FOR	12.460	0.308	0.001	0.003	*	
FAL vs. FOR	9.028	0.244	0.001	0.003	*	
	Df	Sums of Squares	Mean of squares	F-Model	R2	Pr(>F)
Groups	4.000	0.164	0.041	5.873	999.000	0.001
Residuals	65.000	0.455	0.007			
**Reptiles**	F Model	R^2	p-value	p-adjusted	sig	
VFOR vs. FFOR	1.836	0.093	0.017	0.017	*	
VFOR vs. VFAL	2.685	0.088	0.004	0.004	*	
VFOR vs. FAL	3.898	0.122	0.001	0.001	*	
VFOR vs. FOR	5.880	0.246	0.001	0.001	*	
FFOR vs. VFAL	4.665	0.143	0.001	0.001	*	
FFOR vs. FAL	5.764	0.171	0.001	0.001	*	
FFOR vs. FOR	3.842	0.176	0.001	0.001	*	
VFAL vs. FAL	2.601	0.064	0.004	0.004	*	
VFAL vs. FOR	12.627	0.311	0.001	0.001	*	
FAL vs. FOR	12.127	0.302	0.001	0.001	*	
	Df	Sums of Squares	Mean of squares	F-Model	R2	Pr(>F)
Groups	4.000	0.034	0.008	0.928	999.000	0.481
Residuals	65.000	0.589	0.009			
**Amphibians**	F Model	R^2	p-value	p-adjusted	sig	
VFOR vs. FFOR	2.286	0.113	0.011	0.016	*	
VFOR vs. VFAL	1.371	0.047	0.195	0.217		
VFOR vs. FAL	1.532	0.054	0.162	0.203		
VFOR vs. FOR	9.031	0.334	0.001	0.002	*	
FFOR vs. VFAL	8.079	0.224	0.001	0.002	*	
FFOR vs. FAL	7.339	0.214	0.001	0.002	*	
FFOR vs. FOR	6.626	0.269	0.001	0.002	*	
VFAL vs. FAL	0.800	0.021	0.555	0.555		

VFAL vs. FOR	20.104	0.418	0.001	0.002	*	
FAL vs. FOR	17.831	0.398	0.001	0.002	*	
	Df	Sums of Squares	Mean of squares	F-Model	R2	Pr(>F)
Groups	4.000	0.233	0.058	2.808	999.000	0.043
Residuals	64.000	1.327	0.021			
**Butterflies**	F Model	R^2	p-value	p-adjusted	sig	
FOR vs. FFOR	5.881	0.246	0.001	0.002	*	
FOR vs. FAL	8.916	0.242	0.001	0.002	*	
FOR vs. VFOR	6.064	0.252	0.001	0.002	*	
FOR vs. VFAL	10.334	0.270	0.001	0.002	*	
FFOR vs. FAL	4.537	0.139	0.001	0.002	*	
FFOR vs. VFOR	2.696	0.130	0.002	0.003	*	
FFOR vs. VFAL	4.750	0.145	0.001	0.002	*	
FAL vs. VFOR	1.702	0.057	0.012	0.013	*	
FAL vs. VFAL	1.833	0.046	0.006	0.008	*	
VFOR vs. VFAL	1.444	0.049	0.076	0.076		
	Df	Sums of Squares	Mean of squares	F-Model	R2	Pr(>F)
Groups	4.000	0.112	0.028	4.937	999.000	0.002
Residuals	65.000	0.369	0.006			
**Ants**	F Model	R^2	p-value	p-adjusted	sig	
FOR vs. FFOR	2.753	0.133	0.001	0.001	*	
FOR vs. FAL	9.892	0.261	0.001	0.001	*	
FOR vs. VFOR	3.976	0.181	0.001	0.001	*	
FOR vs. VFAL	7.005	0.200	0.001	0.001	*	
FFOR vs. FAL	7.525	0.212	0.001	0.001	*	
FFOR vs. VFOR	1.601	0.082	0.023	0.023	*	
FFOR vs. VFAL	4.760	0.145	0.001	0.001	*	
FAL vs. VFOR	5.429	0.162	0.001	0.001	*	
FAL vs. VFAL	2.909	0.071	0.001	0.001	*	
VFOR vs. VFAL	2.931	0.095	0.001	0.001	*	
	Df	Sums of Squares	Mean of squares	F-Model	R2	Pr(>F)
Groups	4.000	0.008	0.002	0.687	999.000	0.574
Residuals	65.000	0.198	0.003			

Supplementary Table 12: Overview of vanilla statistics for our 30 studied vanilla agroforests depending on vanilla plant or vanilla agroforest

**Variable**	**Mean**	**SD**	**Min**	**Max**
Vanilla age (yrs)/vanilla plant	4.42	3.14	1.28	13.4
Vanilla yield (g)/vanilla plant	30.36	23.93	0	82.23
Vanilla vine length (cm)/vanilla plant	694.06	707.80	0	5198
Planting density (plants/ha)	3284.04	1443.72	1638.79	8519.751
Agroforest age (yrs)	10.8	14.05	3	50
Green vanilla yield kg/ha (2018)	104.61	99.92	0	371.55
Vine length (cm) (mean length /agroforest)	695.30	274.63	219.89	1263.82
Vanilla agroforest size (ha)	0.66	0.56	0.25	3.01

Supplementary Table 13: Determinants of vanilla yield in 30 vanilla agroforests. Model fits of linear mixed effect model (LMM) of vanilla yield (sqrt-transformed) depending on environmental and management variables with all terms significant at <0.05. Significant p-values highlighted in bold. Chisq-values based on based on one-sided Chi-square tests of model comparison with *anova* functon = χ^2 of full model vs. full model excluding each predictor.

	**Vanilla yield (kg/ha)**	
**Variables**	χ^2	p-value
Canopy closure (%)	0.659	0.417
Soil characteristics (PC1)	3.069	0.080
Slope (°)	0.527	0.468
Landscape forest cover (%)	0.587	0.444
Understory vegetation cover (%)	0.030	0.863
Elevation (m)	2.004	0.157
Planting density (no/ha)	17.993	**<0.001**
Pollination (hrs/ha)	6.645	**0.010**
Vanilla vine length (cm)	18.511	**<0.001**
Vanilla plant age (yrs)	0.027	0.869

Supplementary Table 14: Determinants of vanilla yield (kg/ha) in 30 vanilla agroforests (final model). All predictor variables were scaled. Supplementary Table hows analysis of variance results (two-sided t-tests use Satterthwaite's method) for final linear mixed model. Significant p-values (<0.05) are highlighted in bold. Marginal R^2 is the variance explained by fixed effects while conditional R^2 is the variance explained by fixed and marginal effects together.

**Response variable**	**Parameter**	**Estimate**	**Df**	**Std. Error**	**p-value**
Vanilla yield (kg/ha)	Intercept	9.005	8.681	0.804	<0.001
	Vanilla planting density (no/ha)	2.901	20.794	0.415	**<0.001**
	Pollination labour input (hrs/ha)	0.897	22.621	0.469	0.069
	Vanilla vine length (cm)	2.650	19.528	0.393	**<0.001**
	Delta Marginal R^2	0.688			
	Delta Conditional R^2	0.883			

Supplementary Table 15: Model fits of glmmTMB model of tree, herbaceous plant, bird, amphibian, reptile, butterfly and ant richness depending on environmental and management variables using single term deletion with the *anova* function from the R stats package with all terms significant at <0.05. Significant p-values highlighted in bold. Chi-square values based on one-sided Chi-square tests displayed as χ^2.

	Trees		Herbaceous plants		Birds		Amphibians		Reptiles		Butterflies		Ants	
**Variables**	χ^2	p-value	χ^2	p-value	χ^2	p-value	χ^2	p-value	χ^2	p-value	χ^2	p-value	χ^2	p-value
Canopy closure (%)	5.374	**0.020**	0.888	0.346	0.009	0.923	1.373	0.241	6.477	**0.011**	0.052	0.819	3.497	0.062
Soil characteristics (PC1)	3.456	0.063	4.741	0.029	2.311	0.129	1.171	0.279	0.097	0.755	5.090	**0.024**	0.344	0.558
Slope (°)	0.541	0.462	0.090	0.764	0.003	0.953	4.404	**0.036**	0.285	0.593	0.456	0.499	0.941	0.332
Landscape forest cover (%)	4.900	**0.027**	1.433	0.231	0.542	0.462	5.696	**0.017**	0.437	0.509	2.899	0.089	0.770	0.380
Understory vegetation cover (%)	1.540	0.215	0.395	0.530	0.822	0.365	0.002	0.964	3.851	**0.050**	4.388	**0.036**	0.121	0.728
Elevation (m)	0.824	0.364	6.670	**0.010**	0.612	0.434	0.170	0.681	2.763	0.096	1.797	0.180	1.780	0.182
Planting density (no/ha)	9.325	**0.002**	4.006	**0.045**	2.080	0.149	0.817	0.366	0.003	0.958	0.606	0.436	0.118	0.731
Pollination (hrs/ha)	0.156	0.693	0.056	0.812	0.309	0.578	1.671	0.196	2.578	0.108	0.127	0.722	1.041	0.308
Vanilla vine length (cm)	10.034	**0.002**	3.461	**0.063**	1.164	0.281	2.367	0.124	11.156	**0.001**	1.108	0.293	2.065	0.151
Vanilla plant age (yrs)	0.104	0.747	2.171	0.141	0.467	0.494	1.773	0.183	0.242	0.623	0.034	0.855	0.736	0.391

Supplementary Table 16: Model fits of glmmTMB model of endemic tree, endemic herbaceous plant, endemic bird, endemic amphibian, endemic reptile, endemic butterfly and endemic ant richness depending on environmental and management variables using single term deletion with the *anova* function from the R stats package with all terms significant at <0.05. Significant p-values highlighted in bold. Chi-square values based on one-sided Chi-square tests displayed as χ^2.

	**Endemic trees**		**Endemic herbaceous plants**		**Endemic birds**		**Endemic amphibians**		**Endemic reptiles**		**Endemic butterflies**		**Endemic ants**	
**Variables**	χ^2	p-value	χ^2	p-value	χ^2	p-value	χ^2	p-value	χ^2	p-value	χ^2	p-value	χ^2	p-value
Canopy closure (%)	0.002	0.968	6.240	**0.012**	0.050	0.823	1.169	0.280	5.781	**0.016**	0.553	0.457	9.348	**0.002**
Soil characteristics (PC1)	14.106	**0.000**	2.367	0.124	3.870	**0.049**	3.442	0.064	4.188	**0.041**	5.345	**0.021**	1.927	0.165
Slope (°)	3.478	0.062	2.123	0.145	0.004	0.949	4.723	**0.030**	0.333	0.564	0.000	0.993	3.608	0.058
Landscape forest cover (%)	13.278	**0.000**	6.328	**0.012**	0.167	0.683	4.307	**0.038**	0.013	0.909	0.867	0.352	6.330	**0.012**
Understory vegetation cover (%)	3.697	0.055	0.043	0.835	5.007	**0.025**	0.000	0.988	2.632	0.105	4.354	**0.037**	0.418	0.518
Elevation (m)	2.155	0.142	3.593	0.058	2.412	0.120	0.045	0.832	0.166	0.684	0.377	0.539	2.064	0.151
Planting density (no/ha)	14.304	**0.000**	12.092	**0.001**	0.007	0.931	1.191	0.275	0.741	0.389	0.047	0.828	0.345	0.557
Pollination (hrs/ha)	0.033	0.857	2.862	0.091	0.713	0.398	3.349	0.067	0.603	0.438	1.577	0.209	3.996	**0.046**
Vanilla vine length (cm)	24.324	**0.000**	0.001	0.973	1.156	0.282	1.342	0.247	4.919	**0.027**	0.809	0.368	3.420	0.064
Vanilla plant age (yrs)	1.215	0.270	0.017	0.898	2.215	0.137	1.599	0.206	0.008	**0.008**	0.148	0.701	0.006	0.937

Supplementary Table 17: Determinants of species richness in 30 vanilla agroforests (27 vanilla agroforests for tree richness). Estimates of predictor coefficients from the best generalized linear mixed model. Model selection was performed by single term deletion using the anova function from the R stats package with all terms significant at <0.05. Chi-square and Df results are based on analysis of deviance results (one-sided Type II Wald chi-square tests). The p-values are based on a two-sided t-test testing whether the mean is different from 0. Significant p-values (<0.05) are highlighted in bold. Marginally significant values are written in italic (0.050 <= p < 0.100). Marginal R^2 is the variance explained by fixed effects while conditional R^2 is the variance explained by fixed and marginal effects together.

Taxa	Parameter	χ^2	Df,Df_{resid}	Estimate	Std. Error	p-value
Trees	Intercept			2.768	0.110	<0.001
	Landscape forest cover (%)	13.249	1.000	0.270	0.074	**<0.001**
	Planting density (no/ha)	5.498	1.000	-0.155	0.066	**0.019**
	Vanilla vine length (cm)	15.363	1.000	-0.221	0.056	**<0.001**
	Canopy closure (%)	12.043	1.000	0.284	0.082	**<0.001**
	Delta Marginal R^2					0.670
	Delta Conditional R^2					0.884
Herbaceous plants	Intercept			2.991	0.058	<0.001
	Elevation (m)	26.610	1.000	0.283	0.055	**<0.001**
	Soil characteristics (PC1)	3.800	1.000	0.111	0.057	*0.051*
	Planting density (no/ha)	1.374	1.000	0.067	0.058	0.241
	Delta Marginal R^2					0.099
	Delta Conditional R^2					0.224
Amphibians	Intercept			1.498	0.069	<0.001
	Slope (°)	5.319	1.000	-0.125	0.054	**0.021**
	Landscape forest cover (%)	3.477	1.000	-0.097	0.052	*0.062*
	Delta Marginal R^2					0.099
	Delta Conditional R^2					0.224
Reptiles	Intercept			2.011	0.071	<0.001
	Vanilla vine length (cm)	5.470	1.000	-0.166	0.071	**0.019**
	Canopy closure (%)	5.853	1.000	0.178	0.074	**0.016**
	Understory vegetation cover (%)	0.888	1.000	0.072	0.077	0.346
	Delta Marginal R^2					0.310
	Delta Conditional R^2					0.328
Butterflies	Intercept	2.355	0.075	2.355	0.075	<0.001
	Soil characteristics (PC1)	0.011	1.000	0.175	0.069	**0.011**
	Understory vegetation cover (%)	0.077	1.000	-0.127	0.072	*0.077*
	Delta Marginal R^2					0.200
	Delta Conditional R^2					0.365

Supplementary Table 18: Determinants of endemic species richness in 30 vanilla agroforests (27 vanilla agroforests for endemic tree richness). Estimates of predictor coefficients from the best generalized linear mixed model. Model selection was performed by single term deletion using the anova function from the R stats package with all terms significant at <0.05. Chi-square and Df results are based on analysis of deviance results (one-sided Type II Wald chi-square tests). The p-values are

based on a two-sided t-test testing whether the mean is different from 0. Significant p-values (<0.05) are highlighted in bold. Marginal R^2 is the variance explained by fixed effects while conditional R^2 is the variance explained by fixed and marginal effects together.

**Taxa**	**Parameter**	χ^2	**Df,Df_{resid}**	**Estimate**	**Std. Error**	**p-value**
Endemic Trees	Intercept			1.371	0.194	<0.001
	Soil characteristics (PC1)	13.073	1	-0.486	0.134	**<0.001**
	Landscape forest cover (%)	59.906	1	0.827	0.107	**<0.001**
	Vanilla vine length (cm)	28.468	1	-0.553	0.104	**<0.001**
	Planting density (no/ha)	12.259	1	-0.337	0.096	**<0.001**
	Delta Marginal R^2					0.853
	Delta Conditional R^2					1
Endemic herbaceous plants	Intercept			1.022	0.069	<0.001
	Landscape forest cover (%)	13.724	1	0.236	0.064	**<0.001**
	Canopy closure (%)	3.444	1	0.145	0.078	*0.063*
	Planting density (no/ha)	7.861	1	0.176	0.063	**0.005**
	Delta Marginal R^2					0.241
	Delta Conditional R^2					NA
Endemic Birds	Intercept			0.150	0.179	0.402
	Soil characteristics (PC1)	6.58	1	-0.504	0.196	**0.010**
	Understory vegetation cover (%)	6.60	1	0.460	0.179	**0.010**
	Delta Marginal R^2					0.273
	Delta Conditional R^2					0.273
Endemic Amphibians	Intercept			1.335	0.075	<0.001
	Slope (°)	2.812	1	-0.111	0.066	*0.094*
	Landscape forest cover (%)	0.869	1	-0.059	0.064	0.351
	Delta Marginal R^2					0.047
	Delta Conditional R^2					0.129
Endemic Reptiles	Intercept			1.661	0.073	<0.001
	Canopy closure (%)	5.679	1	0.136	0.057	**0.017**
	Soil characteristics (PC1)	5.157	1	0.133	0.059	**0.023**
	Vanilla vine length (cm)	3.331	1	-0.096	0.052	*0.068*
	Delta Marginal R^2					0.001
	Delta Conditional R^2					0.638
Endemic Butterflies	Intercept			1.876	0.044	<0.001
	Understory vegetation cover (%)	3.472	1	-0.092	0.049	*0.062*
	Soil characteristics (PC1)	5.294	1	0.109	0.047	**0.021**
	Delta Marginal R^2					0.033
	Delta Conditional R^2					NA
Endemic Ants	Intercept			1.381	0.096	<0.001

	Canopy closure (%)	12.075	1	0.387	0.111	**0.001**
	Landscape forest cover (%)	6.459	1	0.214	0.084	**0.011**
	Pollination (hrs/ha)	1.991	1	0.129	0.091	0.158
	Delta Marginal R^2					0.532
	Delta Conditional R^2					0.532

Supplementary Table 19: Icon attribution for Figure 1 & 2

**Name**	**Author**	**Link**
Neobatrachia<br>(Amphibian icon in Fig. 1 & 2)	Public Domain	http://phylopic.org/image/c07ce7b7-5fb5-484f-83a0-567bb0795e18/
Dicrurus leucophaeus<br>(Bird icon in Fig. 1 & 2)	Ferran Sayol	http://phylopic.org/image/69c936f5-7ed7-4c72-9081-ac860f1e89d1/
Leptanillini<br>(Ant icon in Fig. 1 & 2)	Public Domain	http://phylopic.org/image/45ccedd3-d5cb-42e8-b158-7e027ab1ff22/
Platanus occidentalis L.<br>(Tree icon in Fig. 1 & 2)	Michele M Tobias	http://phylopic.org/image/806a6ae9-28a0-4dc6-beeb-f6129a44f10e/
Mediodactylus kotschyi<br>(Reptile icon in Fig. 1 & 2)	Public Domain	http://phylopic.org/image/6487de8d-0382-4ba7-b587-5fa2c1119f08/
Compositae<br>(Herbaceous plant icon in Fig. 1 & 2)	Public Domain	http://phylopic.org/image/39335c0c-f879-4df6-90d5-e4f65d90d04e/
Papilionini<br>(Butterfly icon in Fig. 1 & 2)	Public Domain	http://phylopic.org/image/f21829d7-6dfc-4b2f-991c-e16181c24d29/

Supplementary Table 20: Mean, standard deviation, min and max value of all environmental and management variables used for analysis depending on land-use history (VFAL=Fallow-derived vanilla, VFOR=Forest-derived vanilla)

**Parameter**	**Land-use history**	**mean**	**SD**	**min**	**max**
Elevation (m)	VFAL	155.500	185.627	25.000	644.000
Elevation (m)	VFOR	187.700	246.795	20.000	819.000
Slope (°)	VFAL	8.596	4.758	1.050	19.010
Slope (°)	VFOR	7.758	5.944	2.620	17.990
Canopy closure (%)	VFAL	49.161	20.654	9.352	78.811
Canopy closure (%)	VFOR	72.176	12.653	51.362	88.215
Planting density (plants/ha)	VFAL	3535.897	1524.107	2132.987	8519.751
Planting density (plants/ha)	VFOR	2780.331	1178.714	1638.788	5775.310
Vanilla plant age	VFAL	3.798	1.837	1.278	7.886
Vanilla plant age	VFOR	5.667	4.694	1.568	13.395
Soil characteristics (PC1)	VFAL	0.498	2.078	-3.616	4.433
Soil characteristics (PC1)	VFOR	-0.996	0.938	-2.334	0.961
Pollination labour input (hrs/ha)	VFAL	340.620	226.731	52.414	824.690
Pollination labour input (hrs/ha)	VFOR	232.179	196.052	62.163	667.951

Understory vegetation cover (%)	VFAL	74.621	26.360	11.775	100.000
Understory vegetation cover (%)	VFOR	78.241	17.919	49.263	99.875
Forest cover (%)	VFAL	11.296	13.328	0.000	42.056
Forest cover (%)	VFOR	43.209	28.276	0.000	85.845
Vanilla vine length (cm)	VFAL	676.227	266.541	308.853	1263.824
Vanilla vine length (cm)	VFOR	733.450	301.026	219.889	1263.583
Vanilla yield (kg/ha)	VFAL	105.721	113.060	0.000	371.552
Vanilla yield (kg/ha)	VFOR	102.398	71.935	22.970	247.113

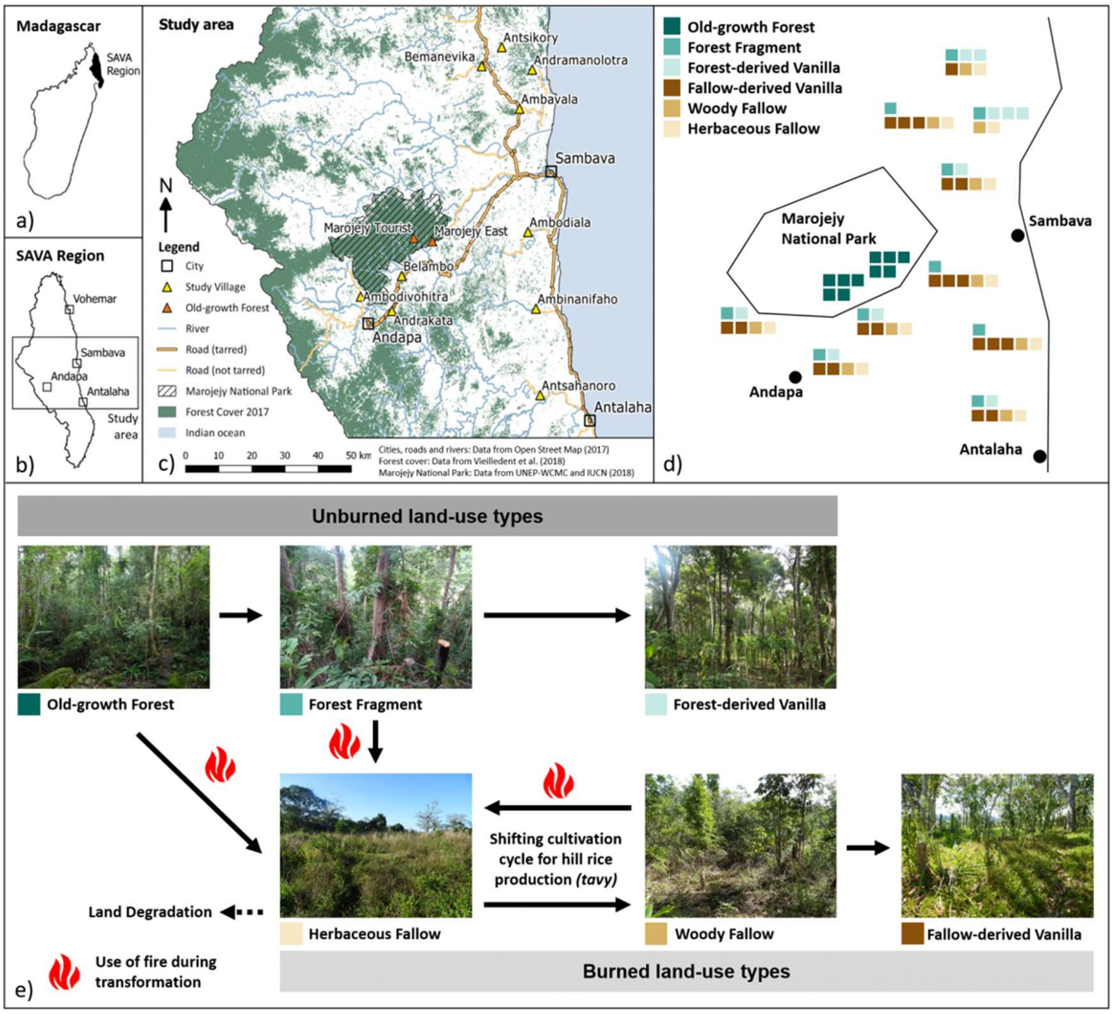

Supplementary Figure 1: Study design overview. a) The island of Madagascar in the Indian Ocean off the coast of East Africa with the SAVA region indicated. b) SAVA region. c) Study area with forest cover in 2017 [26] roads, rivers, and the three main cities Sambava, Antalaha, and Andapa as well as the 10 study villages and Marojejy National Park with its two sampling sites therein. d) Schematic overview of distribution of land-use types across 10 villages and two sampling sides in Marojejy National Park. e) Overview of transformation pathways of unburned and burned land-use types in the study region.

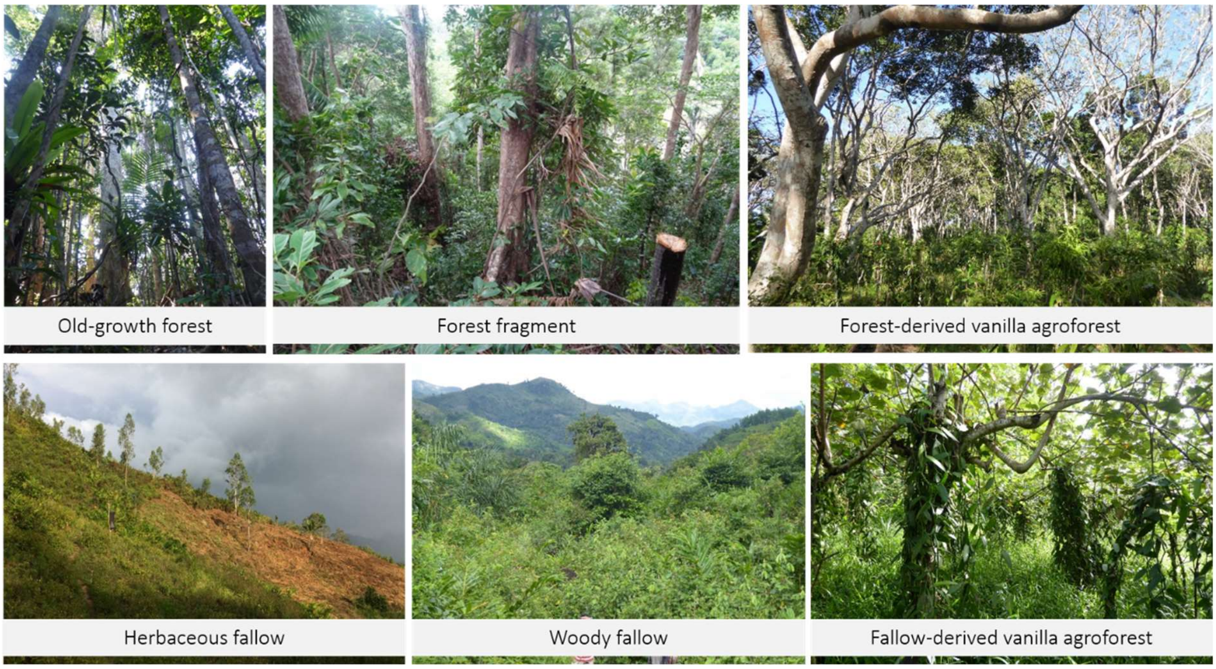

Supplementary Figure 2: Exemplary photos of sampled land-use types: Old-growth forest, Forest fragment, Forest-derived vanilla agroforest, Herbaceous fallow, Woody fallow, Fallow-derived vanilla agroforest (from top left to bottom right).

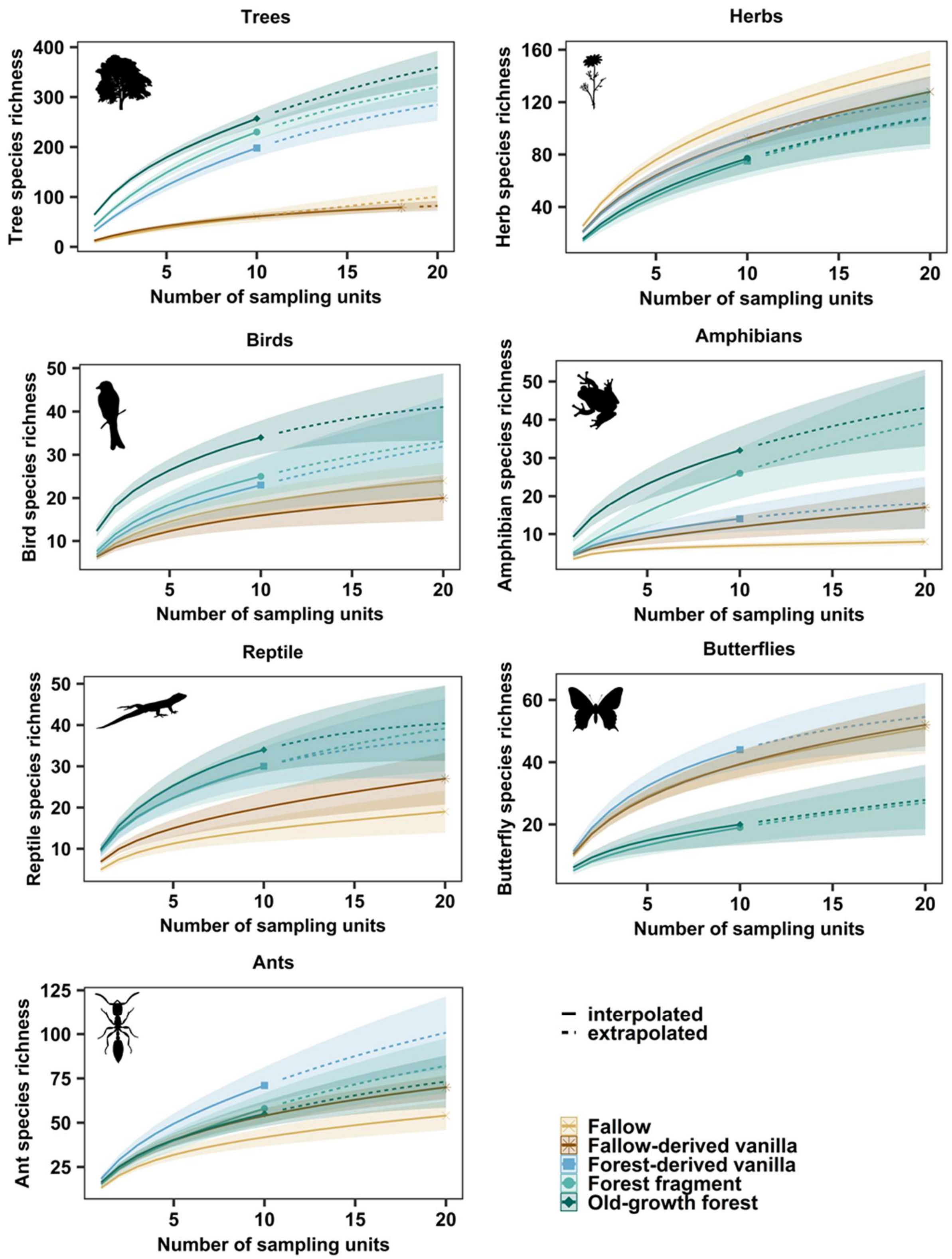

Supplementary Figure 3: Species accumulation curves for all 5 land-use types, shown by the extrapolation curves based on species richness per sampling unit (Hill number q = 0). The solid line represents the interpolated total species number per sampling unit, whereas the dashed line represents the extrapolated total species number per sampling unit. The shaded region represents the 95% confidence intervals and were obtained using a bootstrap method based on 20 replications. The solid dots/quadrats/triangles/crosses represent the reference samples. Non-overlapping of the

confidence intervals represents a significant difference between two or more land-use types. See icon attribution in Supplementary Table 19.

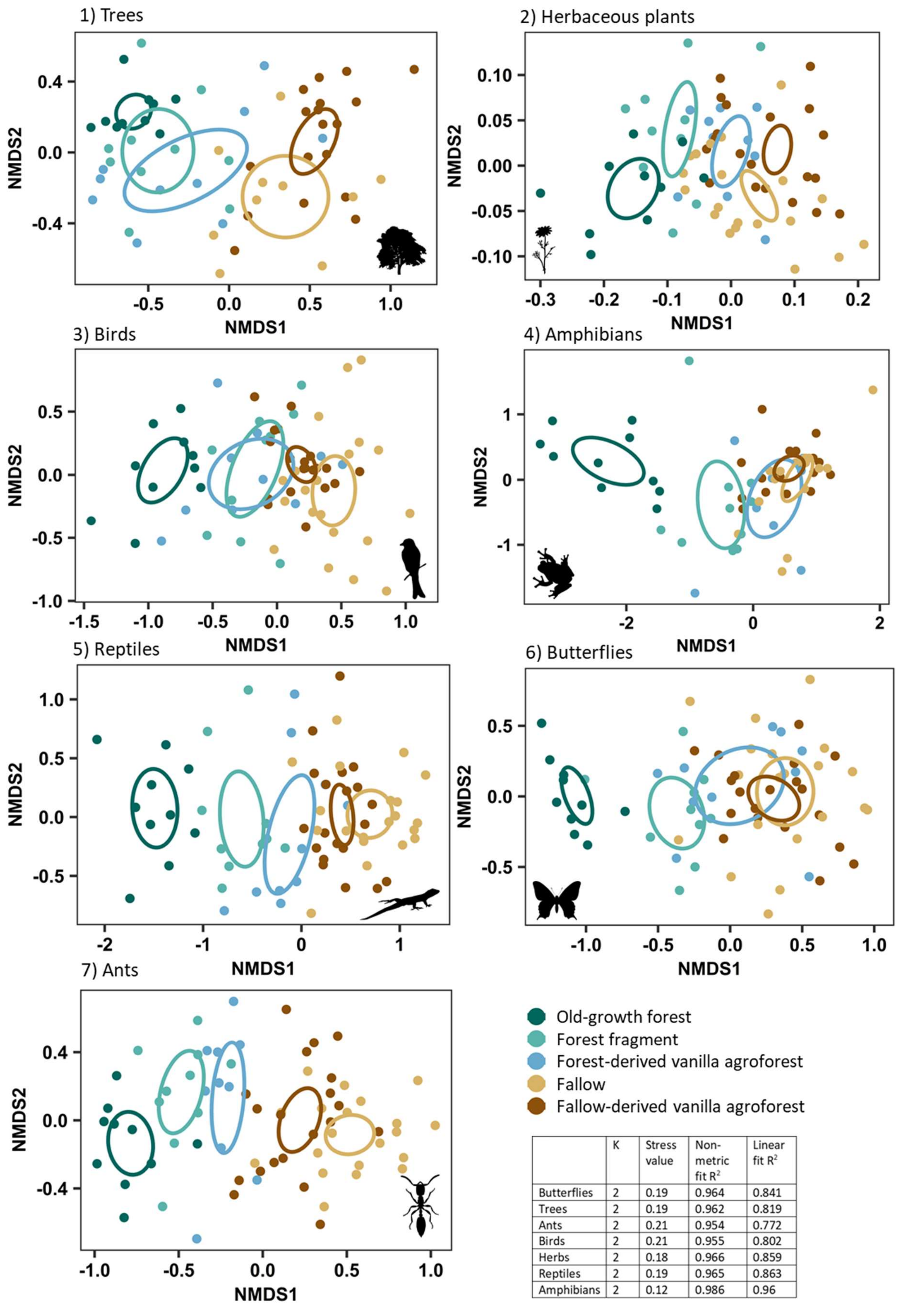

	K	Stress value	Non-metric fit R^2	Linear fit R^2
Butterflies	2	0.19	0.964	0.841
Trees	2	0.19	0.962	0.819
Ants	2	0.21	0.954	0.772
Birds	2	0.21	0.955	0.802
Herbs	2	0.18	0.966	0.859
Reptiles	2	0.19	0.965	0.863
Amphibians	2	0.12	0.986	0.96

Supplementary Figure 4: Species composition of the five land-use types as revealed by non-metric multidimensional scaling (NMDS) based on Jaccard dissimilarity. See icon attribution in Supplementary Table 19.

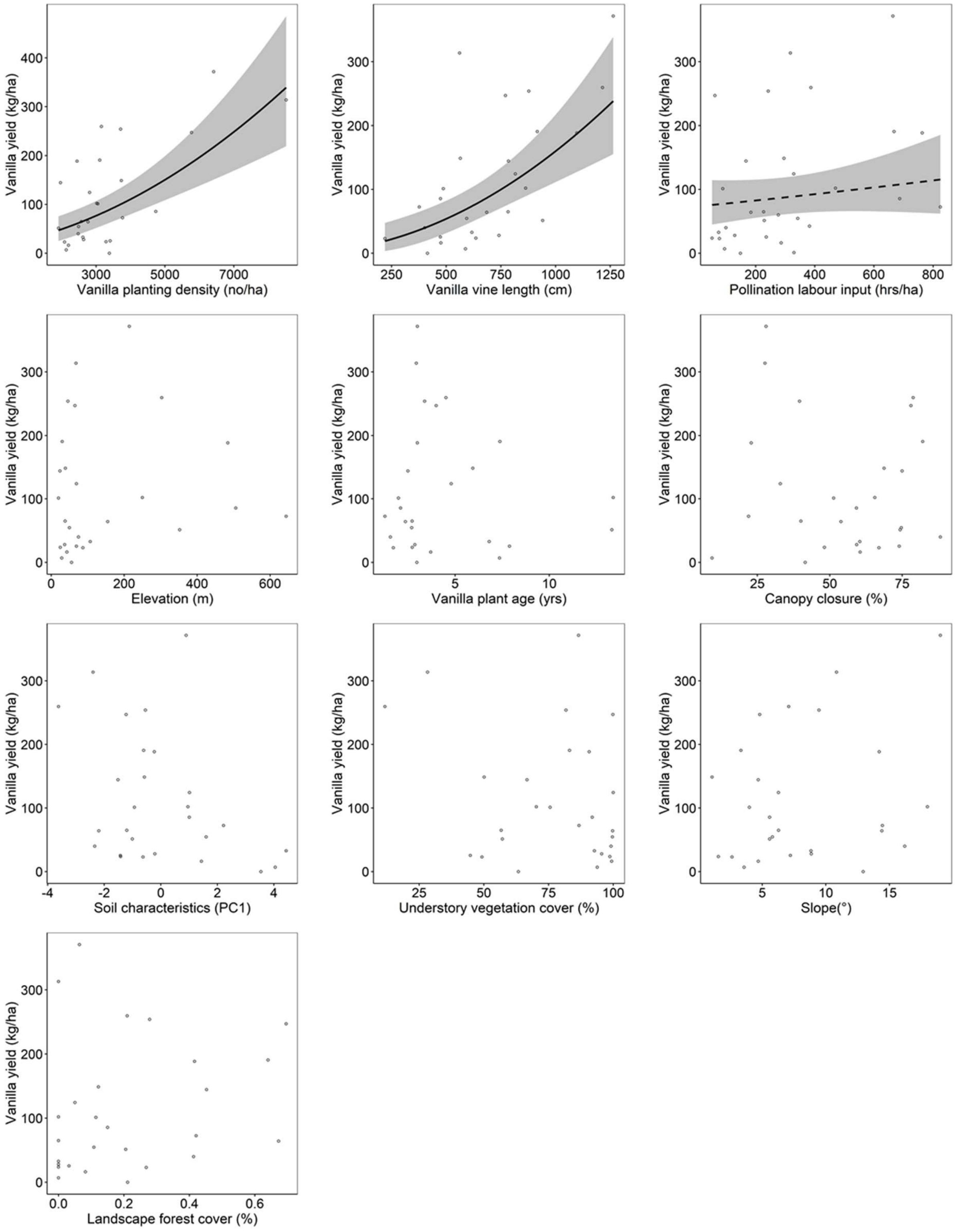

Supplementary Figure 5: Vanilla yield kg/ha depending on planting density, vanilla vine length, pollination labour input, elevation, vanilla plant age, canopy closure, soil characteristics, understory

vegetation cover, slope and landscape forest cover. We used a linear mixed effect model with yield sqrt-transformed. Dots are raw data and solid and dashed lines indicate statistically significant ($p < 0.050$) and marginally significant effects ($0.050 <= p < 0.100$), respectively. Trend lines show average values of the back-transformed model predictions of the final model after using likelihood ratio tests using maximum likelihood estimation, shaded areas indicate 95% confidence intervals.

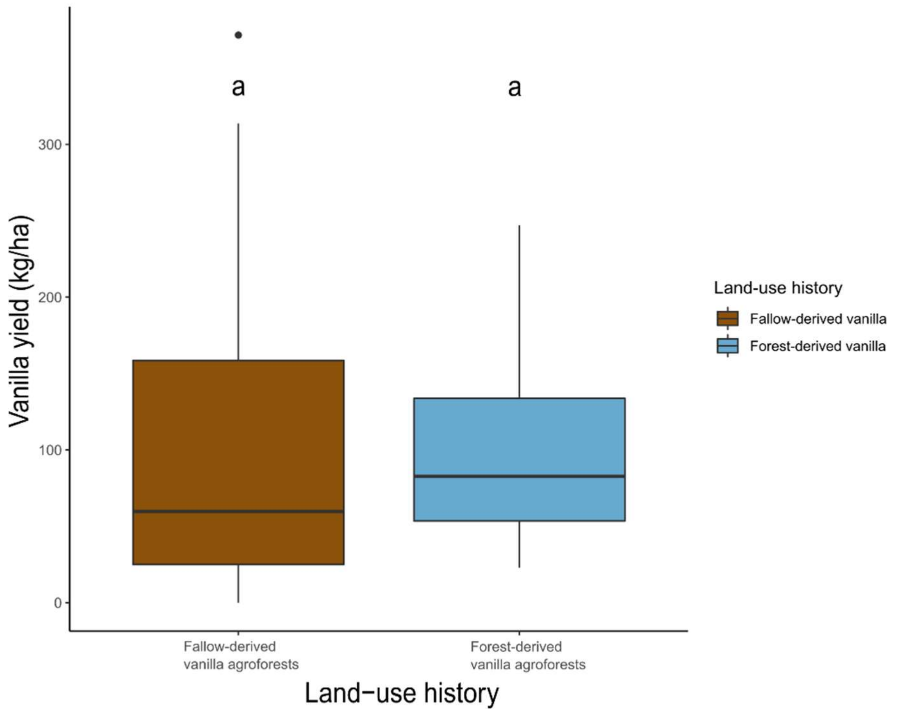

Supplementary Figure 6: Vanilla yield (kg/ha) of 30 vanilla agroforests (n=20 fallow-derived and 10 forest-derived agroforests) depending on land-use history. Linear mixed model with square-root transformed yield depending on land-use history with village as random effect. Multiple comparisons of means: VFOR-VFAL: E=0.7719; SE=1.9407; P=0.691. Shown are boxplots of plot-level endemic mean normalized richness (**A**) and endemic species richness of seven taxa individually (**B-H**) in old-growth forest (FOR), forest fragment (FF), forest-derived vanilla agroforest (VFOR), fallow (FAL) and fallow-derived vanilla agroforest (VFAL) (n=10 for FOR, FF and VFOR & n=20 for FAL and VFAL). The line inside the boxplot represents the median of each land-use type. The lower and upper boundaries of the boxplot show the 25th-75th percentiles of the observational data, respectively and the whiskers show the 1.5 interquartile range. Outliers are shown as dots.

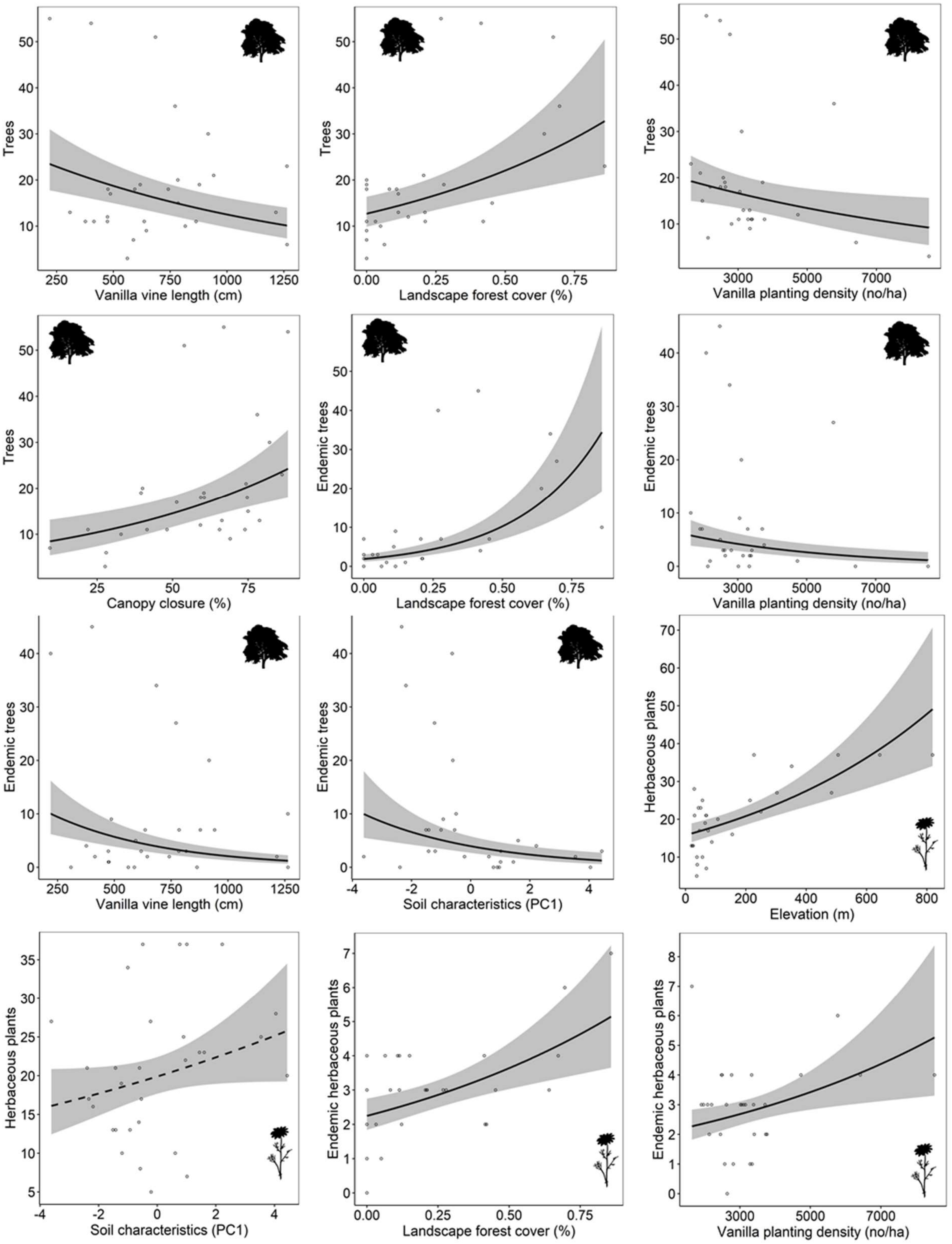
Trees
Vanilla vine length (cm)
Landscape forest cover (%)
Vanilla planting density (no/ha)
Canopy closure (%)
Endemic trees
Soil characteristics (PC1)
Herbaceous plants
Elevation (m)
Endemic herbaceous plants

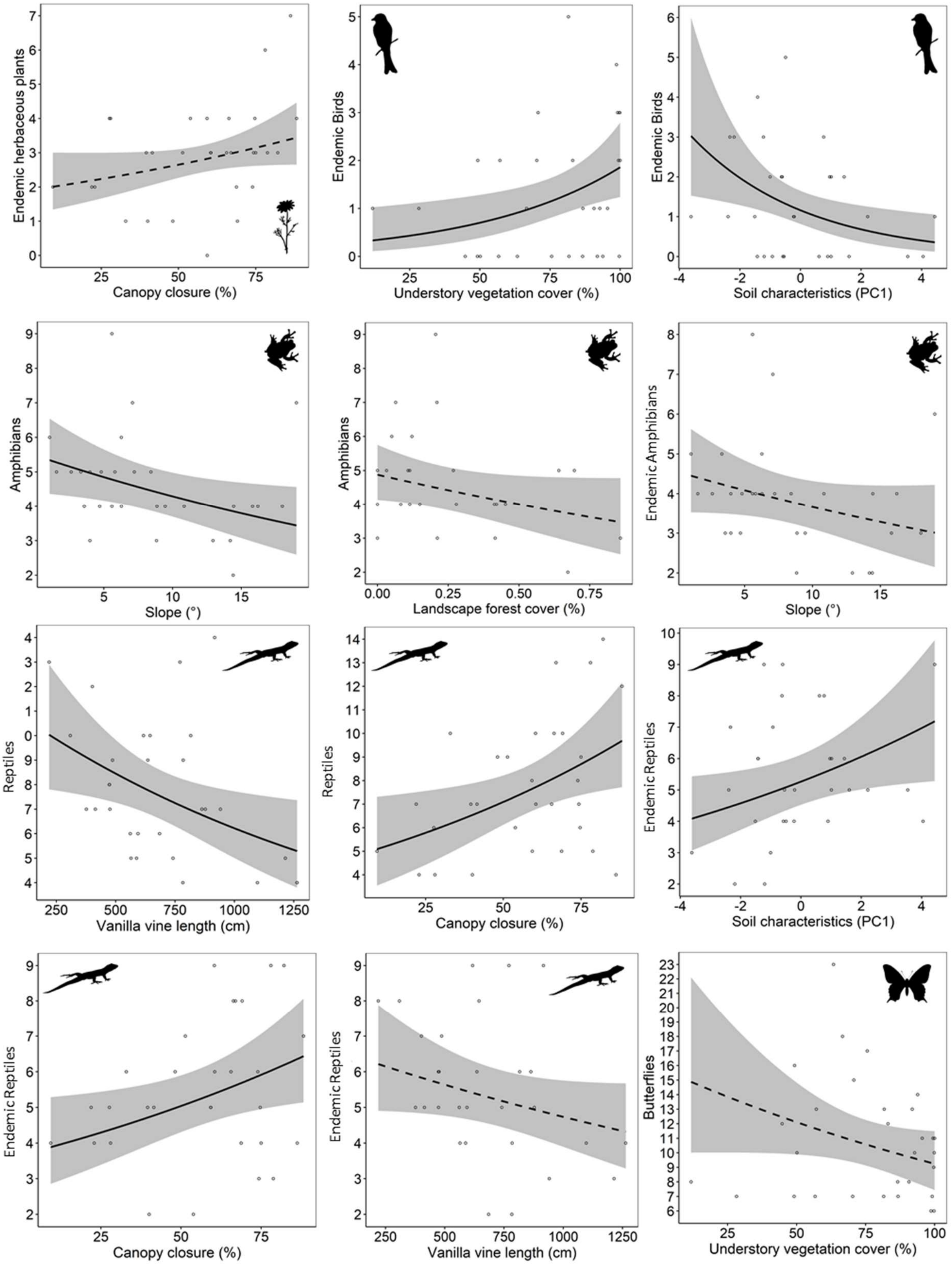
Endemic herbaceous plants
Canopy closure (%)
Endemic Birds
Understory vegetation cover (%)
Endemic Birds
Soil characteristics (PC1)
Amphibians
Slope (°)
Amphibians
Landscape forest cover (%)
Endemic Amphibians
Slope (°)
Reptiles
Vanilla vine length (cm)
Reptiles
Canopy closure (%)
Endemic Reptiles
Soil characteristics (PC1)
Endemic Reptiles
Canopy closure (%)
Endemic Reptiles
Vanilla vine length (cm)
Butterflies
Understory vegetation cover (%)

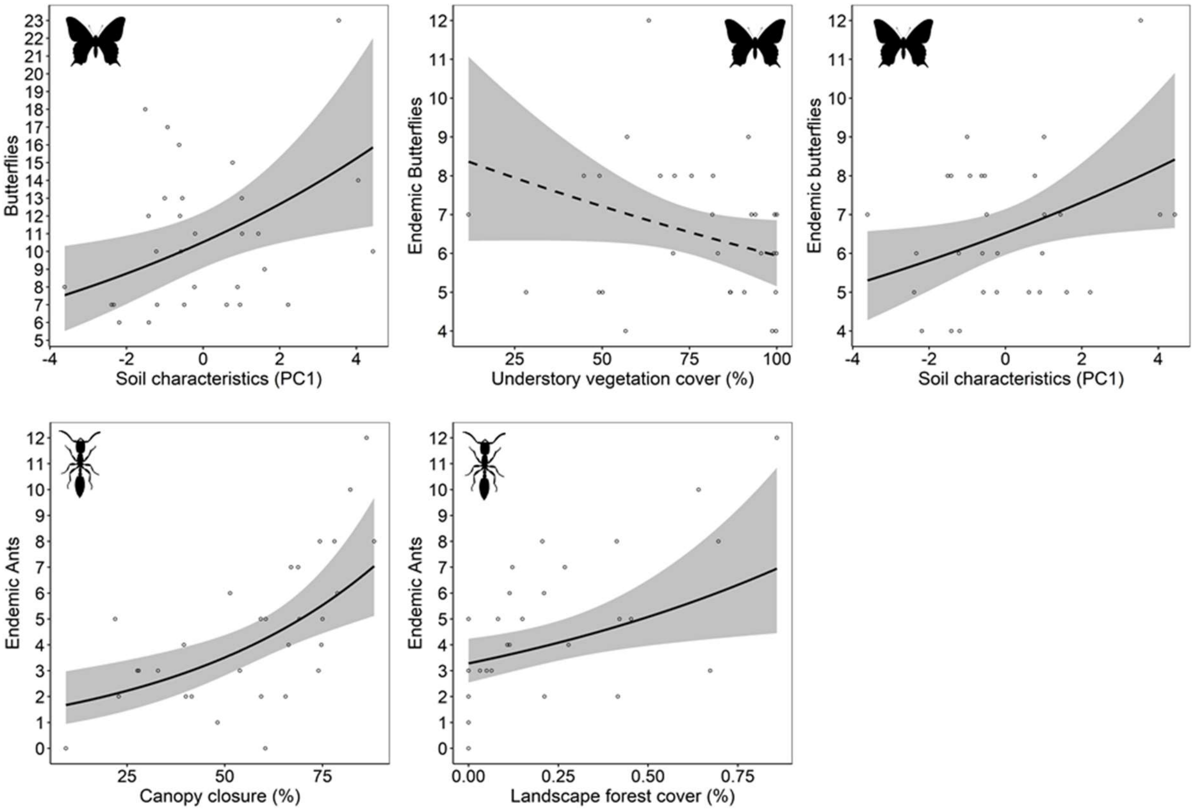

Supplementary Figure 7: (Endemic) species richness of seven different taxa depending on planting density, vanilla vine length, pollination labour input, elevation, vanilla plant age, canopy closure, soil characteristics, understory vegetation cover, slope and landscape forest cover. We used glmmTMB models. Dots are raw data and solid (statistically significant, P<0.05) or dashed (marginally significant, 0.05<p<0.1) trend lines show average values of back-transformed model predictions of the final model after using likelihood ratio tests using maximum likelihood estimation, shaded areas indicate 95% confidence intervals. See icon attribution in Supplementary Table 19.

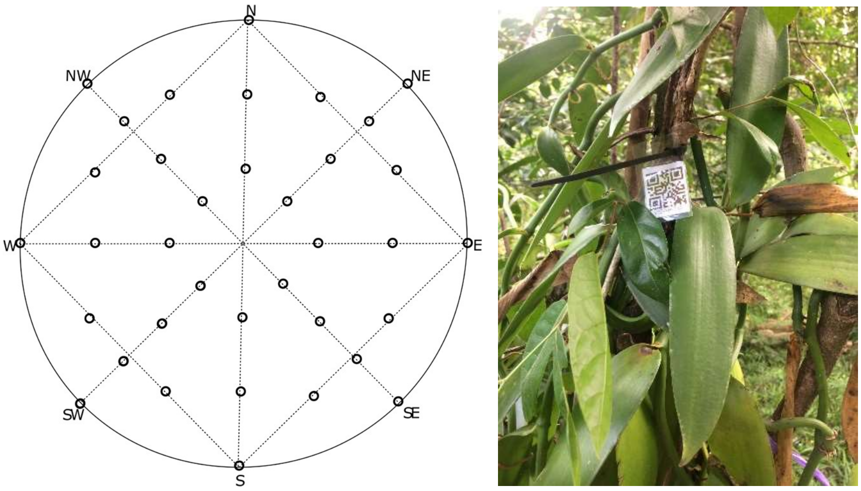

Supplementary Figure 8: Design on the left shows the distribution of 36 vanilla *pieds* on a 25-meter radius plot in a vanilla agroforest (*pied* = combination of a vanilla vine and minimum one support tree). Photo on the right shows unique barcode label on vanilla *pied*.

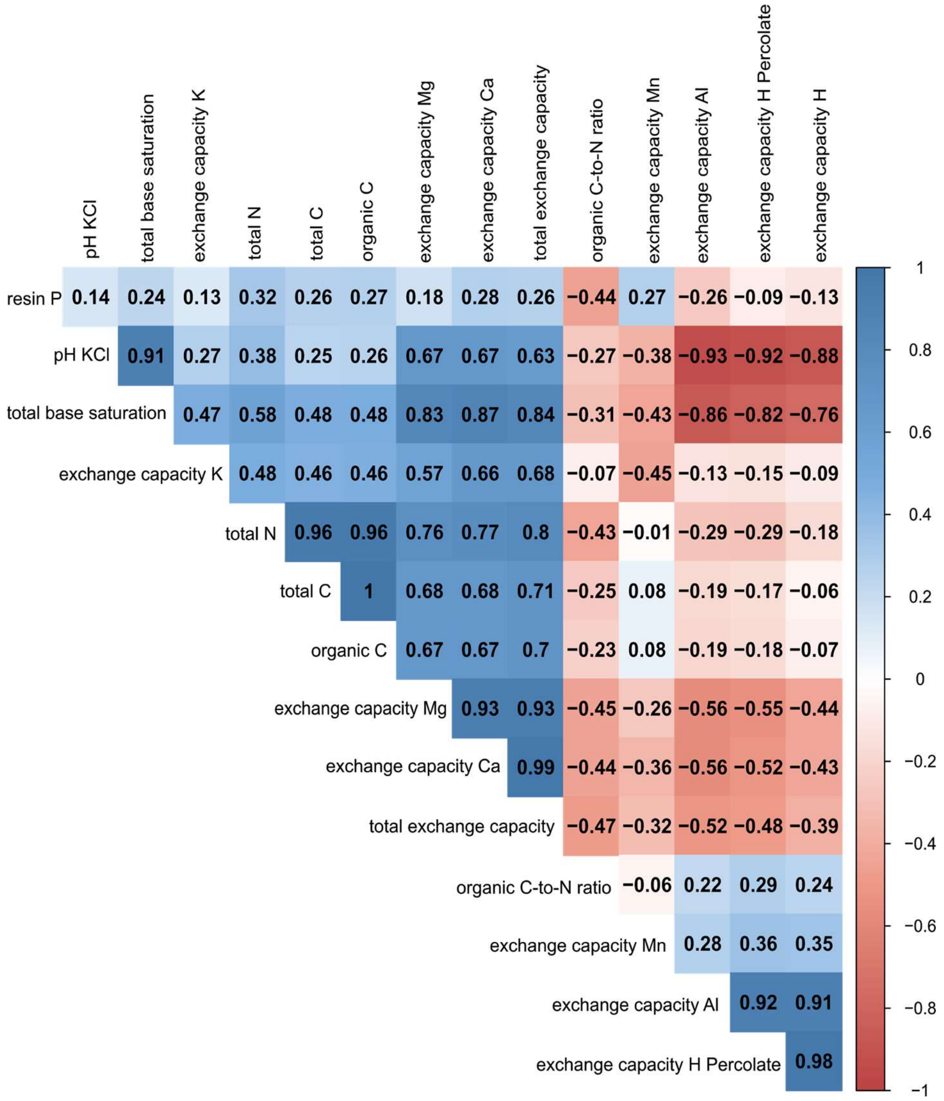

Supplementary Figure 9: Correlation matrix for all pairs of soil variables. Positive correlations are displayed in blue and negative correlations in red color. Color intensity is proportional to the correlation coefficients.

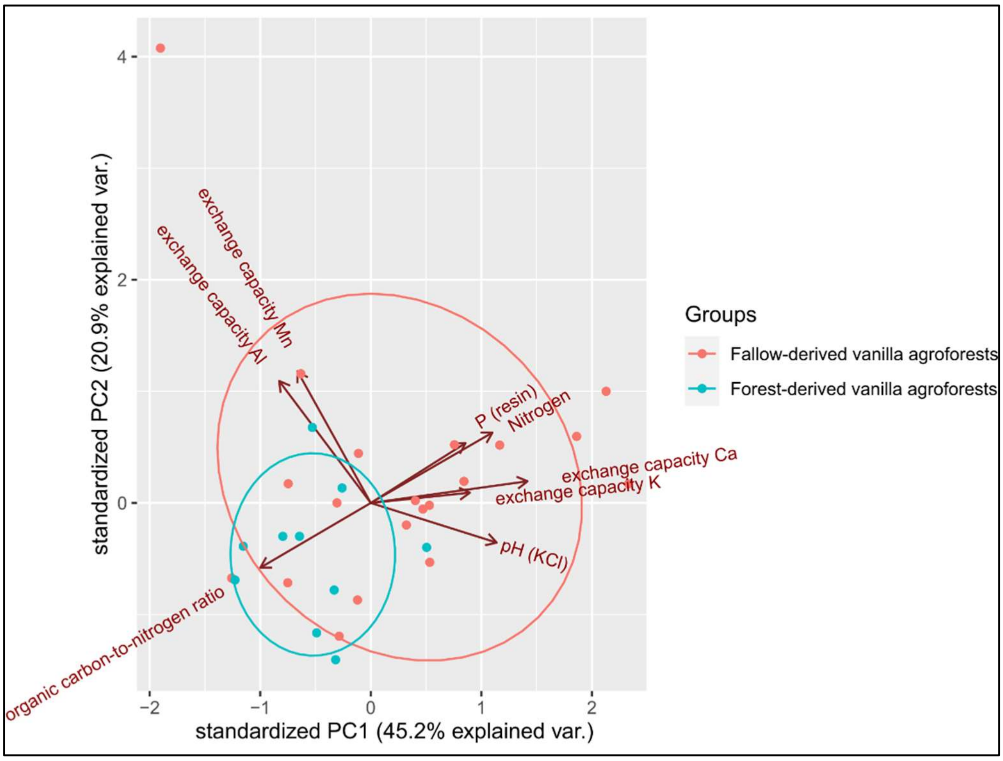

Supplementary Figure 10: PCA based on eight soil characteristics including calcium, aluminum, pH(KCl), potassium, magnesium, nitrogen, phosphorus and carbon-to-nitrogen ratio. PC1 explained 45.2% and PC2 20.9% of the variation. The red ellipse groups variation of soil characteristics in fallow-derived vanilla agroforests and the blue ellipse groups the variation of soil characteristics in forest-derived vanilla agroforests.

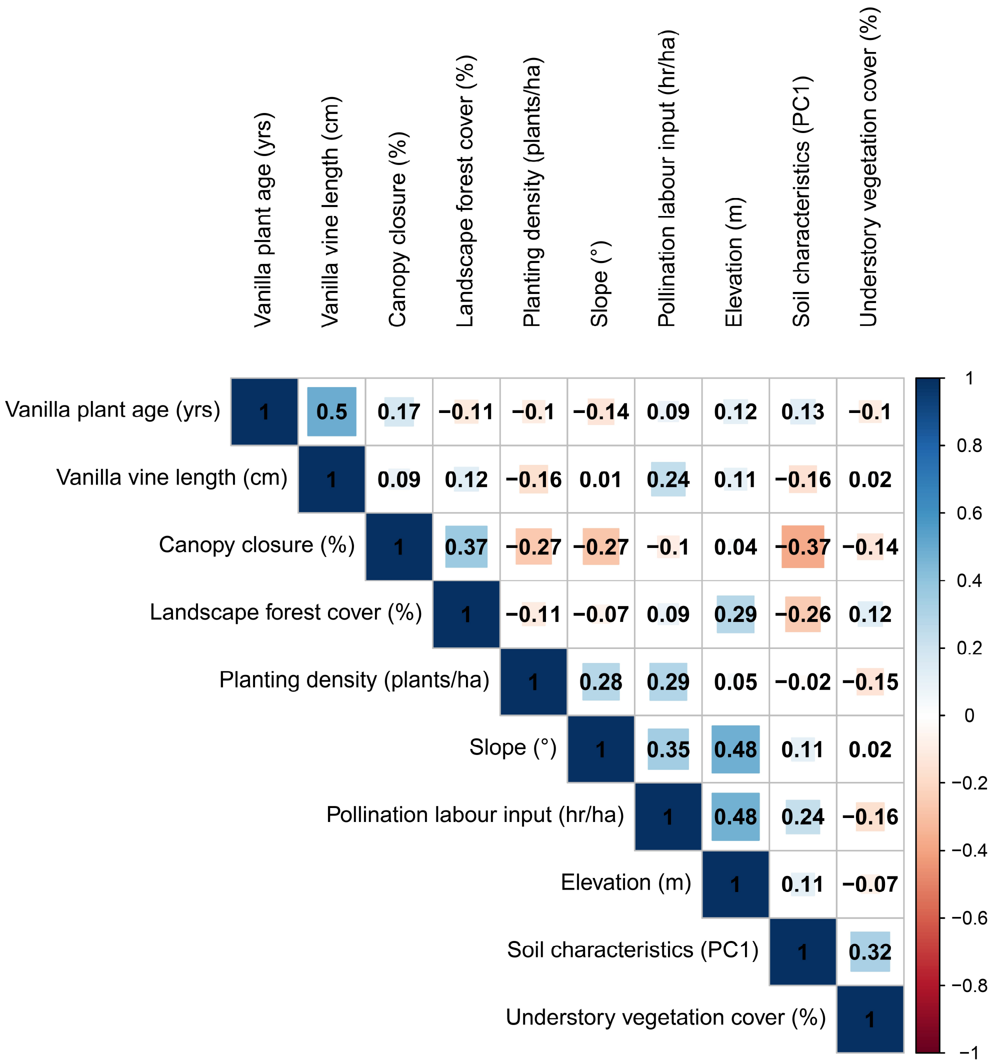

Supplementary Figure 11: Correlation matrix for all pairs of environmental and management variables. Positive correlations are displayed in blue and negative correlations in red color. Color intensity is proportional to the correlation coefficients.

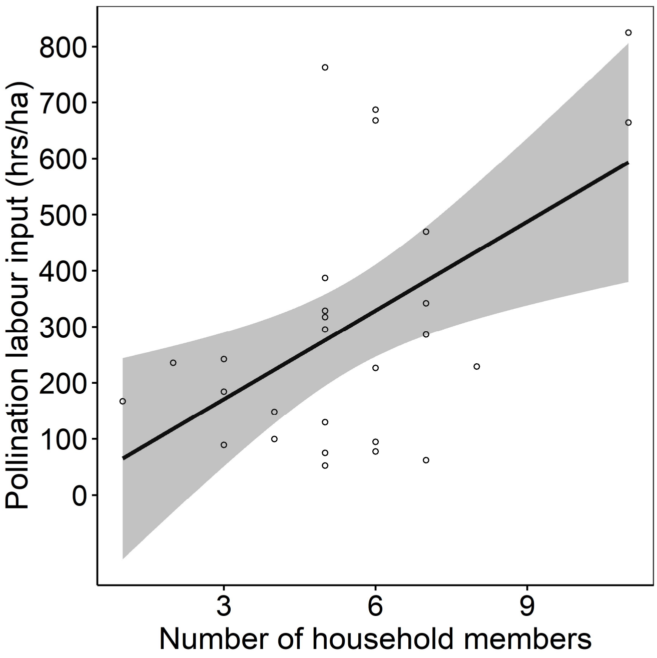

Supplementary Figure 12: Pollination labour input (hrs/ha) depending on the number of household members of 30 households owning the 30 vanilla agroforests of this study using linear regression (p-value=0.005, estimate=52.82, SE=17.38). The p-value is based on a t-test testing whether the mean is different from 0. Dots are raw data and solid (statistically significant, p-value<0.05) trend lines show average values of the back-transformed model prediction including a 95% confidence interval.
