# Supplementary material for: Win-win opportunities combining high yields with high multi-taxa biodiversity in tropical agroforestry
Source: Nat Commun. 2022 Jul 26;13:4127. doi: 10.1038/s41467-022-30866-8 (PMC9325886; doi:10.1038/s41467-022-30866-8)
Supplement: Supplementary file 2 — Reporting Summary [file 41467_2022_30866_MOESM2_ESM.pdf]

nature research

Corresponding author(s): Wurz, Annemarie (NCOMMS-21-13311-A)

Last updated by author(s): 15/5/22

Reporting Summary

Nature Research wishes to improve the reproducibility of the work that we publish. This form provides structure for consistency and transparency in reporting. For further information on Nature Research policies, see our Editorial Policies and the Editorial Policy Checklist.

Statistics

For all statistical analyses, confirm that the following items are present in the figure legend, table legend, main text, or Methods section.

n/a	Confirmed	
☐	☒	The exact sample size (*n*) for each experimental group/condition, given as a discrete number and unit of measurement
☐	☒	A statement on whether measurements were taken from distinct samples or whether the same sample was measured repeatedly
☐	☒	The statistical test(s) used AND whether they are one- or two-sided<br>*Only common tests should be described solely by name; describe more complex techniques in the Methods section.*
☐	☒	A description of all covariates tested
☐	☒	A description of any assumptions or corrections, such as tests of normality and adjustment for multiple comparisons
☐	☒	A full description of the statistical parameters including central tendency (e.g. means) or other basic estimates (e.g. regression coefficient) AND variation (e.g. standard deviation) or associated estimates of uncertainty (e.g. confidence intervals)
☐	☒	For null hypothesis testing, the test statistic (e.g. *F*, *t*, *r*) with confidence intervals, effect sizes, degrees of freedom and *P* value noted<br>*Give P values as exact values whenever suitable.*
☒	☐	For Bayesian analysis, information on the choice of priors and Markov chain Monte Carlo settings
☒	☐	For hierarchical and complex designs, identification of the appropriate level for tests and full reporting of outcomes
☐	☒	Estimates of effect sizes (e.g. Cohen's *d*, Pearson's *r*), indicating how they were calculated

Our web collection on statistics for biologists contains articles on many of the points above.

Software and code

Policy information about availability of computer code

Data collection	R version 3.6.3, KoboToolbox, ALOS World 3D
Data analysis	R version 3.6.3; Code available here: https://osf.io/ndrxg/?view_only=1bd699c5cda64023963e058254a33eec

For manuscripts utilizing custom algorithms or software that are central to the research but not yet described in published literature, software must be made available to editors and reviewers. We strongly encourage code deposition in a community repository (e.g. GitHub). See the Nature Research guidelines for submitting code & software for further information.

Data

Policy information about availability of data

All manuscripts must include a data availability statement. This statement should provide the following information, where applicable:

- Accession codes, unique identifiers, or web links for publicly available datasets
- A list of figures that have associated raw data
- A description of any restrictions on data availability

The code and data used for this study has been deposited in the Open Science Framework database under accession code: https://osf.io/j54fx/?view_only=1bd699c5cda64023963e058254a33eec
The followig databases have been used for this study:
- AntWeb. Version 8.54.9. California Academy of Science. https://www.antweb.org (2021).
- The Reptile Database. The Reptile Database. Uetz, P. Freed, P. Hošek, J. http://www.reptile-database.org (2019).
- AmphibiaWeb. AmphibiaWeb. University of California, Berkeley, CA, USA https://amphibiaweb.org (2019)
- BirdLife International. BirdLife International: Data Zone. (2018) http://datazone.birdlife.org/home
- Madagascar Catalogue. Catalogue of the vascular plants of Madagascar. http://www.efloras.org/madagascar. (2019).

We provide raw data for the following figures: Figure 1, 2, 3, Supplementary Figure 3 -12

Field-specific reporting

Please select the one below that is the best fit for your research. If you are not sure, read the appropriate sections before making your selection.

☐ Life sciences ☐ Behavioural & social sciences ☒ Ecological, evolutionary & environmental sciences

For a reference copy of the document with all sections, see nature.com/documents/nr-reporting-summary-flat.pdf

Ecological, evolutionary & environmental sciences study design

All studies must disclose on these points even when the disclosure is negative.

Study description	Study objective: 1) assessing the biodiversity value of forest- and fallow-derived vanilla agroforests across seven taxa and by comparing their diversity to old-growth forests and degraded land 2) identifying environmental and management drivers of yields and species richness. We collected data in 10 villages covering all prevalent landuse types as well as old-growth forest (up to 10 km away from primary, secondary or tertiary roads) in north-eastern Madagascar.<br><br>Village selection was done through a stratified random sample as we wanted to sample different village sizes evenly distributed. In each village, we selected one herbaceous fallow, one woody fallow, one forest fragment and three vanilla agroforests summing up to 60 plots within 10 villages. Of the 30 selected vanilla agroforests, 20 vanilla agroforests were established on fallow land and 10 vanilla agroforests were directly established inside forest and thus “forest-derived”. Not in all villages were fallow- and forest-derived vanilla agroforests available, leading to an unbalanced design for vanilla agroforests.<br>Additionally, we studied 10 old-growth forest plots at two sites inside Marojejy National Park, the only place with contiguous low-altitude old-growth forest persisting in our study area. We chose the two old-growth forest sites within the same park as a compromise between low-altitude, maximum distance between the two sites and accessibility.<br><br>We added village/site in all our models as random effect to account for regional differences in species richness or vanilla yield.<br>We investigated the relationship of species richness with vanilla yield in 30 vanilla agroforests by using vanilla yield in interaction with land-use history (fallow vs. forest-derived) as explanatory variable.<br><br>For our assessed species groups we had the following number of assessments: trees (n=68), herbaceous plants (n=70), birds (n=70), amphibians (n=70), reptiles (n=70), butterflies (n=70) and ants (n=70).
Research sample	We studied 7 different taxa across 70 plots including the population of invertebrate (butterfly and ants), the population of vertebrates (amphibian, reptile, birds) and the population of plant (trees and herbs). Trees (n=68; population of all plants with DBH >8cm), herbaceous plants (n=70, population of non-woody plants), birds (n=70), amphibians (n=70), reptiles (n=70), butterflies (n=70; population of all day-flying butterflies) and ants (n=70). We sampled 2 plant taxa, 3 vertebrate taxa and 2 invertebrate taxa to gain a balanced representation of taxonomic groups in our studied land-use types.
Sampling strategy	We chose 10 replicates per land-use type as comprimise between travel time between villages and plots, plot size and survey time needed for all taxa studied.<br><br>In each of the 10 villages we selected three vanilla agroforests, one forest fragment, and two fallows. Overall, we studied 70 plots across 10 villages and 10 plots in one protected old-growth forest (Marojejy National Park). All plots had a minimum distance of 260 m and a mean minimum distance of 794 m (SD=468m) to each other. Plot elevation ranged between 10 and 819 m.a.s.l. (mean =205 m, SD=213 m; Supplementary Table 20).<br><br>Out of our 30 vanilla agroforests, 20 vanilla agroforests were fallow-derived and 10 vanilla agroforests were forest-derived, roughly matching with the proportion of fallow- and forest-derived vanilla agroforests across the study region (70% are fallow-derived vanilla agroforests, 27% are forest-derived vanilla agroforests and 3% of unknown origin). We chose 10 herbaceous as well as 10 woody fallow for the land-use type fallow because fallows generally occur in these different successional stages. We also studied 10 plots at two sites in Marojejy National Park, the only remaining, continuous old-growth forest at low-altitude in our study area. In addition, 10 forest fragments were sampled. Forest fragments were located inside the agricultural landscape and were remnants of once continuous forest; these fragments are frequently used for natural product extraction.
Data collection	Trees were assesed by tree inventory all trees with diameter at breast height >8cm (by Kristina Osen, Thorien Rabemanantsoa, Jean Chrysostome Bevao, Patrice Antilahimena, Marie Rolande Soazafy). The inventory included trees, arborescent palms, herbs, and tree ferns but excluded lianas. We identified tree species with the help of a local tree expert (Chrysostome Bevao) and a taxonomic expert (Patrice Antilahimena) from Missouri Botanical Garden (Antananarivo, Madagascar). Herbaceous plant were assessed on vegetation suplots (by Jeannie Marie Estelle Raveloaritiana). We sampled herbaceous plants in eight subplots of 4 m2 each (32 m2 overall) between September 2018 and December 2019. In each subplot, we assessed vascular plant species without apparent wood at maturity. Birds were assessed using pointcouns (by Dominic Martin, Eric Rakotomalala, Saskia Dröge, Rouvah Andriafanomezantsoa). We sampled birds during two 40 min point counts per plot with two observers per point count following a commonly used standardized method. For calculating bird species richness per plot, we disregarded observations only in flight and outside the 25-m-radius of plots. We sampled amphibians and reptiles using repeated time-standardized search walks for 45 min by two observers (by

Thio Rosin Fulgence, Romual Randriamanantena). We visited each plot both during the day and at night both during the driest and the wettest period. We systematically walked the circular plot in a zig-zag pattern always starting from the West part toward North, East, South, and end in West to avoid counting twice the same individual during observation in one of the plot. We actively checked microhabitats to detect individuals hiding therein (e.g., individuals hiding under rocks, in leaf axils, tree barks, tree holes, leaf litter, or deadwood). When encountering an individual, we stopped the standardized search time and identified the individual. Butterflies were sampled using fruit traps and time-standardized netting (by Annemarie Wurz, Anjaharinony Rakotomalala, Evrard Benasoavina,Theudy Alexis). We baited fruit traps with fermented bananas and deployed the cylindrical nets for 24 hours. Before deployment, we fermented bananas for 48 hours in an air-tight container. On each plot, we installed a total of 8 fruit traps. We deployed four fruit traps at 16.6 m distance from the plot center in the four main cardinal directions and the other four fruit traps at 20 m distance from the center in the four intercardinal directions. We caught butterflies with a fruit trap with a 20 cm Cone Opening (90 cm long hanging 1.5 m above the ground. On plots without trees, we installed fruit traps on a support stick (in rice paddy and herbaceous fallow). During the 30 minutes time-standardized netting, we caught butterflies within an imaginary 2 m wide box to each side of the net while walking at a slow and steady speed in a zig-zag to equally cover the plot area. Ants were sampled using pitfalls and bait traps (by Anjaharinony A. N. A. Rakotomalala,Theudy Alexis). To do so, we established five sampling stations per plot: one at the plot center, and four at 16 m distance from the plot center; one in each cardinal direction. We then set bait and pitfall traps 10 m apart at each sampling station. We baited the bait traps using sardine and sugar on two white flat plastic plates with a diameter of 13 cm and placed the two plates about 5 cm apart. We left the baited traps for 30 minutes before collecting ants for 30 seconds. We buried the pitfall traps (plastic cups of 9 cm top diameter, 11 cm depth, and 6 cm bottom diameter) in the soil and filled them one-third with 70-%-alcohol and a few drops of soapy water. We emptied the pitfall traps after 48 hours and identified ants to species/morphospecies level in the laboratory.

Vanilla yield was assessed pre-harvest (by Annemarie Wurz, Kristina Osen, Dominic Martin, Evrard Benasoavina, Gatien Rasolofonirina, Thorien Rabemanantsoa). We assessed vanilla vine length for all 36 vanilla pieds (same vanilla pieds as used for the yield assessment) on each plot by measuring the total length of the vine from the lowest to the highest part with a measuring stick. We assessed the vanilla yield prior to harvest to ensure an accurate yield assessment due to two reasons: Firstly, vanilla pods are commonly harvested successively due to their differing pollination date and maturity requiring multiple visits over several weeks. Secondly, theft of vanilla pods is commonplace around harvest time. We therefore estimated the weight of the on-plant-hanging vanilla pod by a length-by-width formula. This is possible because vanilla pods only grow in length and width in the first eight weeks of their development.

We assessed pollination labour input by interviewing the plotowners (Fanilo Andrianisaina, Hendrik Hänke, Fenohaja Soavita Babarezoto). Each plot owner filled out pictograms daily over one year giving us information on family labour input in pollination and other agricultural activities, such as weeding, harvesting, curing of vanilla and others.

We (Annemarie Wurz, Kristina Osen, Dominic Martin, Evrard Benasoavina, Gatien Rasolofonirina, Thorien Rabemanantsoa) assessed vanilla plant age by asking the farmer for each of the 36 vanilla pieds per plot.

We (Annemarie Wurz, Kristina Osen, Dominic Martin, Evrard Benasoavina, Gatien Rasolofonirina, Thorien Rabemanantsoa) assessed vanilla vine length for all 36 vanilla pieds (same vanilla pieds as used for the leaf damage and yield assessment) on each plot by measuring the total length of the vine from the lowest to the highest part with a measuring stick.

We (Kristina Osen) used the 30 m-resolution digital surface model "ALOS World 3D" by Japan Aerospace Exploration Agency (JAXA) to assess the mean slope and the mean elevation of each plot. For all values we applied slope correction.

We (Dominic Martin) calculated forest cover in a 250 m radius around each plot centre based on binary forest cover data from 2017 with a 30 m resolution and the R-package raster. We chose this size of radius as a compromise between mobile and immobile taxa.

We (Jeannie Marie Estelle Raveloaritiana, Claudine Bemamy) estimated the vegetation cover (woody and herbaceous plants) visually for the 0-2 m layer in % of five subplots on each plot (located in the plot centre and at 16.6 m from the centre in each cardinal direction) and calculated the mean understory vegetation cover per plot. We did not consider vanilla pieds in the estimation of the understory vegetation cover.

We (Jeannie Marie Estelle Raveloaritiana, Annemarie Wurz, Anjaharinony Rakotomalala, Evrard Benasoavina) took soil samples with a MacFadyen soil corer (5 cm diameter, 295 ml, 0-15 cm depth). We divided the plots into eight subplots, four subplots at 8.3 m distance to the plot centre (inner area) and four subplots at 16.6 m distance to the plot centre (outer area). In total, we collected four cores in the inner and outer area each, resulting in two mixed soil samples per plot. We stored each soil sample in a zip-lock bag until laboratory analysis.

We measured mean canopy closure (Kristina Osen, Thorien Rabemanantsoa, Adriane März) at five subplots of our circular plots by taking hemispherical images with a Nikon D5100 camera. The camera was fixed on a tripod at 2.4 m height above vanilla support trees and understory vegetation. We selected the images with the best contrast of sky and vegetation using the histogram-exposure protocol and calculated canopy closure using a minimum thresholding algorithm.

Timing and spatial scale

We sampled trees on all land-use types except herbaceous fallows between September 2018 and January 2019. We sampled herbaceous plants in eight subplots of 4 m2 each between September 2018 and December 2019.

We sampled birds during two 40 min point counts per plot with two observers per point count. For birds, we conducted one round of point counts between September-December2017 and a second one between August and December 2018. In old-growth forest plots, we did all point counts in 2018 (August 2018 and December 2018). We sampled amphibians and reptiles by visiting each plot both during the day and at night both during the driest (one nocturnal and one diurnal search between October and December 2017; one nocturnal and one diurnal search between August and December 2018) and the wettest period (one nocturnal and one diurnal search between January and April 2018 or in February 2019). We did so during the driest period and during the wettest period. We sampled butterflies with fruit traps and time-standardized netting between August and December 2018. We sampled ground-foraging ants using bait and pitfall traps. We conducted the sampling in all villages between October and December 2017, and in the

old-growth forest in August and December 2018. We performed a longitudinal survey with the plot owners of our 30 vanilla agroforests for the pollination labour input from October 2017 to October 2018.

Villages in which data was collected are located up to 60km north, south and west from Sambava (north-eastern Madagascar). We sampled in the region's seven most prevalent land-use types during the year's driest period between August and November 2018 when climate and weather are rather constant (Plot assessibility was given as most roads are passable during this time; Assessment of birds, butterflies and ants is only feasible during non-rainy conditions). For amphibians and reptiles we sampled also in the wettest period, as activity of amphibians and reptiles peaks during that time.
Species data was collected on plots with a radius of 25m. We chose this size as a compromise between plot size and survey time needed for all taxa studied. We established our circle plots in a homogeneous area of the land-use type or forest.

Data exclusions

No data was excluded

Reproducibility

Data and R code available on Open Source Framework

Randomization

No samples/participants or specimens were allocated into groups.

We selected 10 villages based on the 60 villages selected within the Diversity Turn in Land Use Science project (Hänke et al., 2018). Among the 60 villages, we considered all villages without coconut plantations, with less than 40% water (river, sea and lakes) and with forest fragments and shifting cultivation present within a 2 km radius around the village. Two of these 17 villages overlapped within the 2 km radius of the villages, thus we randomly selected one of them, resulting in 14 villages. We visited these 14 villages in a randomized order and stopped after we found 10 villages which fulfilled the necessary criteria (all land use types present, willing to participate). In each of the 10 villages we selected three vanilla agroforests and two fallows. Overall, we studied 50 plots across 10 villages and 10 plots in one protected forest (Marojejy National Park).

We assessed vanilla plant data (yield, vine length etc.) on 36 vanilla pieds on each of 30 circular vanilla plots. We defined one vanilla pieds (foot in French) as the combination of a vanilla vine and minimum one support tree. The 36 vanilla pieds were evenly selected across the circle plot based on a sampling protocol to ensure comprehensive and unbiased sampling. We chose vanilla pieds independent of age, length or health condition.

Blinding

Blinding was not relevant to our study as species were assessed in a standardized manner on each plot following sampling protocols.

Did the study involve field work? ☒ Yes ☐ No

Field work, collection and transport

Field conditions

All plots were situated in northeastern Madagascar in the SAVA region (Supplementary Figure S1). The natural vegetation is tropical lowland rainforest, but deforestation rates are high. The region is a global but also national biodiversity hotspot with high levels of endemism. Forest loss is mainly driven by slash-and-burn agriculture for hill rice cultivation. The region is characterized by a warm and humid climate with an annual rainfall of 2255 mm and a mean annual temperature of 23,9 °C (mean value of 60 plots extracted from CHELSA climatology).

Location

Sampling took place around the main cities of Sambava (-14.268900, 50.164449), Antalaha (-14.905075, 50.280410) and Andapa (-14.659301, 49.651765) in north-eastern Madagascar. All plots had a minimum distance of 260 m and a mean minimum distance of 794 m (SD=468m) to each other. Plot elevation ranged between 10 and 819 m.a.s.l. (mean =205 m, SD=213 m).

We provide a table with plot coordinates on OSF (https://osf.io/j54fx/?view_only=1bd699c5cda64023963e058254a33eec). Plot coordinates (longitude / latitude) have been reduced to three digits to protect the privacy of land owners.

Access & import/export

We collected data under research permits N°100/17/MEEF/SG/DGF/DSAP/SCB.Re, N°163/17/MEEF/SG/DGF/DSAP/SCB.Re, N°18/18/MEEF/SG/DGF/DSAP/SCB.Re, and N°254/18/MEEF/ SG/DGF/DSAP/SCB.Re granted by the Ministry for Water, Ecology and Forest (MEEF), Antananarivo. We labelled all samples of ants (alcohol samples), herbaceous plants (dried herbarium samples), trees (dried herbarium samples), butterflies (dried specimens), soil (dried soil samples), amphibians and reptiles (DNA samples) with plotcode and sampling location. We thus provided exact number and identity of samples (e.g. CITES species) to the Ministry for Water, Ecology and Forest (MEEF) in Sambava. MEEF visually inspected all specimens and issued a transport permit. Export permits were issued for butterflies, soil, amphibians and reptiles only, as all plants were identified and stored at the herbarium in Tsimbazaza, Antananarivo and ants at Madagascar Biodiversity Centre, Antananarivo. Export permits were issued by the MEEF in Antananarivo in collaboration with the University of Antananarivo (Department Entomology, Animal Biology and Plant Biology). All samples and exports permits were checked and stamped by MEEF at the airport before departure.

Disturbance

Small soil cavities made by soil sampling and pitfall traps were closed after the sample extraction. To inform each plot owner and village members about the sampling procedure, meetings were held before and after sampling for each taxon. Sampling was done with approval of each plot owner. For our tree inventory, leaf and fruit samples needed for identification were taken without injuring the tree. For herbaceous plant identification, only one sample per species was taken per plot.

Reporting for specific materials, systems and methods

We require information from authors about some types of materials, experimental systems and methods used in many studies. Here, indicate whether each material, system or method listed is relevant to your study. If you are not sure if a list item applies to your research, read the appropriate section before selecting a response.

Materials & experimental systems	
n/a	Involved in the study
☒	☐ Antibodies
☒	☐ Eukaryotic cell lines
☒	☐ Palaeontology and archaeology
☐	☒ Animals and other organisms
☐	☒ Human research participants
☒	☐ Clinical data
☒	☐ Dual use research of concern

Methods	
n/a	Involved in the study
☒	☐ ChIP-seq
☒	☐ Flow cytometry
☒	☐ MRI-based neuroimaging

Animals and other organisms

Policy information about studies involving animals; ARRIVE guidelines recommended for reporting animal research

Laboratory animals: The study did not involve laboratory animals.

Wild animals: For amphibian and reptiles, we took DNA samples to determine the species for those individuals that proved difficult to identify using morphological characteristics only. To retrieve a DNA sample, we collected muscle or toe clips as tissue samples, conserved in 90% of alcohol. We stored DNA samples at the Evolutionary Biology laboratory at TU Braunschweig. We also took photos of specimens that we did not identify to species level (ventral, back, and flank view). Until release, we kept them in a ventilated bag to retain moisture. We released all specimens after completing the full-time-standardized search.
For ants, we killed specimens in 70% alcohol by collecting them in pitfall samples or sucking the specimens from bait plates into sample tubes. For butterflies, we used cotton balls soaked with 25% ammoniak acid in a glas to euthanize specimens. Ants and butterflies had to be collected, as unique morphological features required use of microscope, identification key and species verifications by experts.
For all collected specimens, we did not assess sex or age but only identified the species because sex and age were not analysed in our study.

Field-collected samples: Ant specimens were collected between October and December 2017 and between August and December 2018. Ant specimens are either mounted or preserved in alcohol at Madagascar Biodiversity Center, Antananarivo, Madagascar. Butterfly samples were collected between August and December 2018 and are stored in a dry cooler (10°C) at the Department of Crop Science, Agroecology lab in Goettingen. Amphibian and reptile were collected between October and December 2017; August and December 2018; January and April 2018 or in February 2019. The tissue samples of amphibians and reptiles are stored in alcohol at the Evolutionary Biology laboratory at TU Braunschweig.

Ethics oversight: Ethics approval was obtained for this study from the ethics committee of the University of Goettingen (Chair: Prof. Dr. Peter-Tobias Stoll) under the reference number 17./04.22-Wurz. Guidelines of “Good Scientific Practice” by the University of Göttingen were adopted (adapted based on the recommendations of the codex for good scientific practice from the DFG, German Research Foundation; https://www.uni-goettingen.de/en/good+scientific+practice/567647.html).

Note that full information on the approval of the study protocol must also be provided in the manuscript.

Human research participants

Policy information about studies involving human research participants

Population characteristics: We performed a longitudinal survey with the plot owners of our 30 vanilla agroforests from October 2017 to October 2018. The questionnaire was pre-tested with 30 farmers in September 2017. All participants were trained by the four research assistants on how to use pictogram-supported questionnaires. Subsequently, a feedback workshop was held to adapt the pictograms and optimize the entire questionnaire. The pictograms had to be filled every day as a diary. Besides pollination labour input, we assessed the time spent on plantation establishment, planting, weeding, pruning, plantation safeguarding, harvesting, preparing (fermenting, drying, sorting), and selling of vanilla (not considered in this analysis). Every fortnight, trained assistants visited farmers to collect the diary questionnaires. Data entries that appeared unusual were verified with the farmers by the assistants. The diary questionnaires included questions on family labour input for pollination as well as other agricultural activities, such as weeding, harvesting, curing of vanilla, and others.
The household head who filled the pictograms received 10,000 Ariary (roughly 2,50 €) per month. Pictograms are drawings made by a local artist which visually describe each of the working steps of vanilla cultivation (e.g. planting vanilla vine, weeding plantation, pollination). The amount of compensation was recommended as a reasonable compensation by locally experienced Malagasy project members. The sum was handed out by the local research assistants at the end of each month. All participants of the surveys were informed that participation is voluntary, that they can leave the survey anytime, and that all data is anonymized, i.e., no personal data will be published or shared with third parties.

Recruitment: In each of the 10 villages, we held village meetings with the village chief and anyone who was interested in our study. We presented our research objectives and the land-use types we were interested. Subsequently, we made appointments with individual households to visit their plots. Ultimately, we selected three vanilla agroforests with low, medium, and high canopy closure, respectively, covering a within village canopy cover gradient. To refine our vanilla agroforest classification, we used interviews with the plot owners to categorize all vanilla agroforests based on land-use history into fallow- and forest-derived agroforests. The owners of our study plots were the same who participated in the longitudinal study to assess pollination

	labour input over one year.
Ethics oversight	The interview methodology (i.a. informed consent by the test persons as well as the questionnaires) was evaluated by the ethics committee of the University of Goettingen (https://www.uni-goettingen.de/en/534983.html) and complied with their principles of the Higher Education Act of Lower Saxony (NHG) and the constitutionally protected right of academic freedom (Reference number: 17./04.22-Wurz) .

Note that full information on the approval of the study protocol must also be provided in the manuscript.
